# A novel oral formulation of the melanocortin-1 receptor agonist PL8177 resolves inflammation in preclinical studies of inflammatory bowel disease and is gut restricted in rats, dogs, and humans

**DOI:** 10.3389/fimmu.2023.1083333

**Published:** 2023-02-20

**Authors:** John Dodd, Robert Jordan, Marie Makhlina, Keith Barnett, Ad Roffel, Carl Spana, Alison Obr, Priyanka Dhingra, Paul S. Kayne

**Affiliations:** ^1^ Palatin Technologies, Inc., Cranbury, NJ, United States; ^2^ Consulting & Advisory Services – Clinical Pharmacology, ICON plc, Groningen, Netherlands

**Keywords:** PL8177, melanocortin, melanocortin 1 receptor, alpha-melanocyte–stimulating hormone, inflammatory bowel disease, inflammation, pharmacokinetics

## Abstract

**Introduction:**

PL8177 is a potent and selective agonist of the melanocortin 1 receptor (MC1R). PL8177 has shown efficacy in reversing intestinal inflammation in a cannulated rat ulcerative colitis model. To facilitate oral delivery, a novel, polymer-encapsulated formulation of PL8177 was developed. This formulation was tested in 2 rat ulcerative colitis models and evaluated for distribution, *in vivo*, in rats, dogs, and humans.

**Methods:**

The rat models of colitis were induced by treatment with 2,4-dinitrobenzenesulfonic acid or dextran sulfate sodium. Single nuclei RNA sequencing of colon tissues was performed to characterize the mechanism of action. The distribution and concentration of PL8177 and the main metabolite within the GI tract after a single oral dose of PL8177 was investigated in rats and dogs. A phase 0 clinical study using a single microdose (70 µg) of [^14^C]-labeled PL8177 investigated the release of PL8177 in the colon of healthy men after oral administration.

**Results:**

Rats treated with 50 µg oral PL8177 demonstrated significantly lower macroscopic colon damage scores and improvement in colon weight, stool consistency, and fecal occult blood vs the vehicle without active drug. Histopathology analysis resulted in the maintenance of intact colon structure and barrier, reduced immune cell infiltration, and increased enterocytes with PL8177 treatment. Transcriptome data show that oral PL8177 50 µg treatment causes relative cell populations and key gene expressions levels to move closer to healthy controls. Compared with vehicle, treated colon samples show negative enrichment of immune marker genes and diverse immune-related pathways. In rats and dogs, orally administered PL8177 was detected at higher amounts in the colon vs upper GI tract. [^14^C]-PL8177 and the main metabolite were detected in the feces but not in the plasma and urine in humans. This suggests that the parent drug [^14^C]-PL8177 was released from the polymer formulation and metabolized within the GI tract, where it would be expected to exert its effect.

**Conclusion:**

Collectively, these findings support further research into the oral formulation of PL8177 as a possible therapeutic for GI inflammatory diseases in humans.

## Introduction

The melanocortin system is an important part of the immune system in many tissues throughout the body and is upregulated during inflammation (1, 2). Melanocortin receptors are widely distributed throughout various organ systems (eg, central nervous system, cardiovascular and gastrointestinal [GI] systems) and expressed by many cell types, including skin, immune, and endothelial cells (3). Melanocortins comprise a family of 4 peptides: adrenocorticotropic hormone (ACTH) and α-, β-, and γ-melanocyte–stimulating hormones (MSH) (2). α-MSH binding to the melanocortin 1 receptor (MC1R) results in anti-inflammatory effects through inhibition of nuclear factor kappa B (NF-κB) (4, 5) and proinflammatory cytokines such as tumor necrosis factor alpha (TNF-α) (6, 7), upregulation of interleukin (IL)–10 (8), and through switching immune cells from a proinflammatory to a regulatory phenotype (9).

The anti-inflammatory benefits of α-MSH have been demonstrated in numerous animal experimental models and in human cells *in vitro* (2, 10–14). Multiple lines of evidence have also demonstrated a role for the melanocortin system in colitis and suggest the potential for MC1R agonists specifically in the treatment of intestinal inflammation (15). For instance, mice with a frameshift mutation in the MC1R gene experience significantly greater colitis in response to dextran sulfate sodium (DSS) or *Citrobacter rodentium* (a transmissible form of murine colitis) compared with wild-type mice (15). Moreover, α-MSH reduced symptoms of fecal blood and weight loss, and reduced production of TNF-α and nitric oxide in a mouse model of inflammatory bowel disease (IBD) (16).

The MC1R selective agonist PL8177 (Palatin Technologies, Inc, Cranbury, NJ, USA) and its main active metabolite (PL8435) have demonstrated a binding affinity and functional activity that mirrors that of α-MSH when evaluated against MC1R receptors (17–19). In rat and murine preclinical studies, PL8177 and its main metabolite performed similarly to α-MSH in both preventing and reversing intestinal and ocular inflammation (17). Intracolonic administration of PL8177 resulted in improved colonic weight and decreased markers of inflammation compared with vehicle control in a rat model of colitis, and equivalent to the positive control sulfasalazine (17). Consequently, an oral formulation has been developed for the local treatment of ulcerative colitis and other types of IBD. In this formulation, PL8177 is co-precipitated with Eudragit polymers into a homogeneous noncrystalline solid (Evonik, Allentown, PA, USA) to protect it from degradation in the upper GI tract and to release PL8177 in the lower GI tract.

Studies to investigate the potential value of an oral formulation of PL8177 for the treatment of GI tract inflammation were conducted. This report describes the effects of PL8177 in 2,4-dinitrobenzene sulfonic acid (DNBS)- and DSS-induced rat models of colitis and the pharmacokinetic (PK) properties of orally administered polymer-encapsulated PL8177 in animal and human studies.

## Materials and methods

### Ethics

Studies with PL8177 in rat models of colitis were performed by Pharmacology Discovery Services Taiwan, Ltd. (New Taipei City, Taiwan) and the animal care and use protocol was reviewed and approved by the Institutional Animal Care and Use Committee at Pharmacology Discovery Services Taiwan, Ltd. The rat and dog PL8177 distribution studies were performed by ITR Laboratories Canada Inc. (Baie d’Urfe, QC, Canada), and all procedures were approved by their Animal Care Committee and in accordance with local legislation. Animal housing, experimentation, and animal disposal were performed in general accordance with the principles outlined in the *Guide to the Care and Use of Experimental Animals* (Canada) (20) and/or the *Guide for the Care and Use of Laboratory Animals* (USA) (21). The clinical study was performed by PRA Health Sciences (Groningen, Netherlands) and was conducted in accordance with the principles of the Declaration of Helsinki and in compliance with the International Council for Harmonisation E6 Guideline for Good Clinical Practice, and with the EU Clinical Trial Directive (Directive 2001/20/EC). All participants provided written informed consent. The study was approved by the independent, accredited Ethics Committee of Beoordeling Ethiek Biomedisch Onderzoek (Assen, Netherlands).

### Preclinical studies

#### PL8177 in DNBS colitis–induced rat model of colitis

PL8177 was examined in multiple studies using the DNBS-induced colitis (22) model in rats Unless specifically stated otherwise PL8177 always refers to the polymer encapsulated formulation. In all these studies PL8177 capsules or vehicle capsules were administered by oral gavage to male Wistar rats weighing 200 ± 20 g at 24, 12, and 2 hours before and 6 hours after singular intracolonic delivery of DNBS (30 mg in 0.5 mL ethanol 30%). Eight to 12 rats were included in each treatment group. Rats then received twice-daily doses of vehicle or PL8177 for 5 days, through day 7. These studies compared different doses: 50, 100, and 200 µg (Study 1); 10, 20, and 50 µg (Study 2); and 20 and 100 µg (Study 3). In all studies colitis was assessed by damage scores (diarrhea and rectal bleeding) and colon weight gain on day 8.

#### PL8177 in DSS colitis–induced rat model of colitis

**Figure 1:**
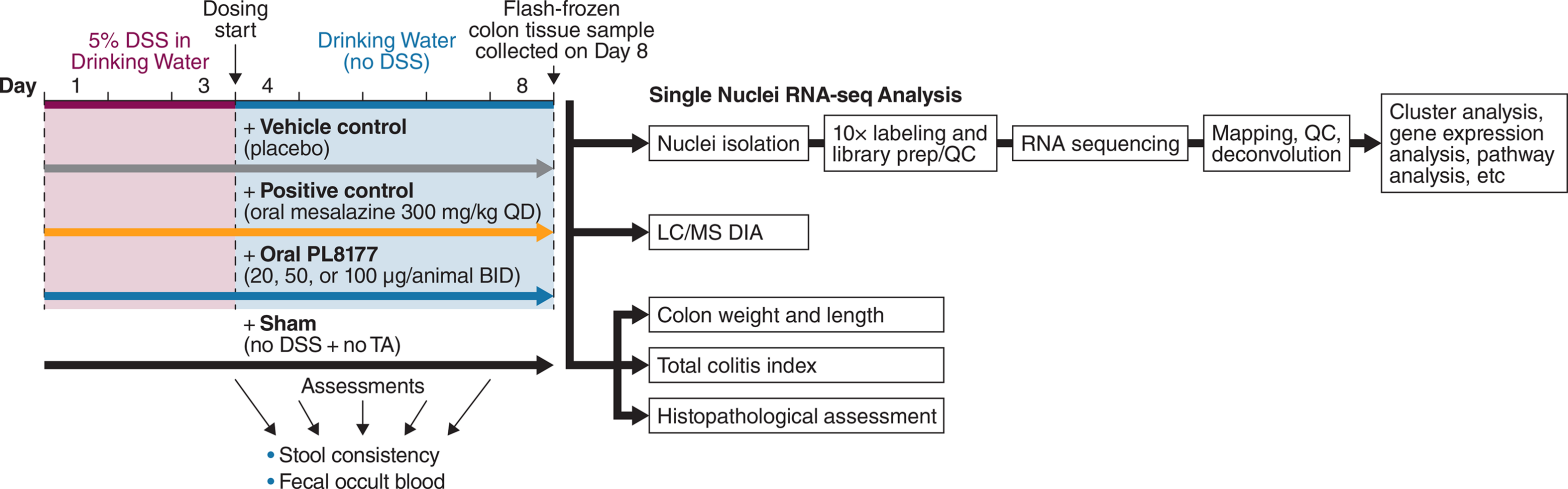
Design of the DSS-induced rat colitis study. BID, twice daily; DSS, dextran sulfate sodium, LC/MS DIA, data-independent acquisition tandem mass spectrometry QC, quality control; QD, once daily; RNA-seq, RNA sequencing; TA, test agent.

This study (n=42) examined the potential curative effect of an oral formulation PL8177 in a DSS-induced colitis rat model (23). Groups of 6 male Wistar rats weighing 200 ± 20 g were used. All animals, except those in the sham group, received 5% DSS in the drinking water for 3 days, from day 1 to day 3, which was then changed to normal drinking water for the following 5 days in the study. The rats in the sham group were given drinking water only. Vehicle control placebo capsules or PL8177 capsules at 20, 50, or 100 μg/animal were administered by oral gavage twice daily at an interval of 6 hours starting on day 4 through day 7, for a total of 4 consecutive days. Mesalazine, which is approved for the treatment of IBD, including ulcerative colitis and Crohn’s disease, was administered orally as a positive control (300 mg/kg once daily) starting on day 4 through day 7 for a total of 4 consecutive days (**Figure 1** **)**. Colitis was assessed by disease activity index (diarrhea and rectal bleeding) during the study and by colon length shortening, colon weight gain, and colon histopathologic assessment after organ harvesting on day 8. At termination on day 8, 24 hours after the last dose, colon tissues were harvested, rinsed, photographed, weighed, and their lengths measured, and then cut into 2 halves 7 cm from the anus. One half of the harvested colon tissue was snap-frozen with liquid nitrogen for cytokine measurement (interferon [IFN]-γ, IL-1β, IL-6, and TNF-α). The other half of the colon tissue was further cut into 3 parts 2.5, 5, and 7 cm from the anus, fixed in 10% formalin, and kept in 70% ethanol for histopathology. The colon length and colon-to-body weight ratio was calculated for each animal according to the formula: colon weight (g)/body weight (g) × 100%. Colon tissues from rats (n=6 per treatment group) were drop-fixed in 4% paraformaldehyde embedded in paraffin, and sectioned at 4 µm. For histologic scoring, colon sections were stained with hematoxylin and eosin. For immunohistochemistry, colon sections were processed for antigen retrieval and immunostained with primary antibody: keratin 18 (K18) (1:400, Bioss, bs-5405R) and with a rabbit-specific biotin-conjugated secondary antibody from HistoWiz (HistoWiz, Inc. Brooklyn, NY). Representative images were taken at 10x magnification and quantification was performed using HistoWiz AI technology counting positive cells versus all nuclei, specifically in the crypts. All analyses were performed by blinded individuals.

Histologic criteria for the analysis of colitis included abnormalities of mucosal architecture, extent of inflammation, erosion or ulceration, epithelial regeneration, and the percentage involvement of the disease process. The scoring was based on the findings of independent observers by examining 3 colon sections from each animal. Scoring was from 0 = none to 4 = severe for each item, except for percentage involvement of disease process, which scored from 1 (1%–25%) to 4 (76%–100%). Total scores for colitis (total colitis index) were added, resulting in a combined histologic score range from 0 to 60 (**Table S1**).

##### Single nuclei RNA sequencing (snRNA-seq) bioinformatics analysis

Nuclei were isolated from 9 flash-frozen rat colon tissue samples and were used to construct 3’ single cell gene expression libraries (Next GEM v3.1) using the 10x Genomics Chromium system (10x Genomics, Pleasanton, CA). Libraries were sequenced with ~150 million read pairs (PE150) per sample on an Illumina NovaSeq sequencing system (Illumina, San Diego, CA). After sequencing, gene counts were generated by Cell Ranger v6.0.1 (10x Genomics) using the rat reference genome *Rattus norvegicus* 6.0.97. Introns were included in the analysis. CellBender (24) was applied to eliminate empty droplets and technical artifacts. Only genes detected in a minimum of 3 cells, cells with at least 200 genes, and cells with <5% of mitochondrial reads were retained. Each sample was normalized using *sctransform* (25), part of the Seurat toolkit v4.0.3 (26). To analyze complete data sets and perform downstream comparative analysis between treatment groups (PL8177 vs sham, PL8177 vs vehicle), we combined 3 PL8177 samples and 3 sham or vehicle samples and used the Seurat integration method (27) to remove batch effects and enable identification of shared population across samples within each treatment group.

##### snRNA-seq data set dimension reduction and cell type identification

For the top variable genes, principal component analysis, Uniform Manifold Approximation and Projection (UMAP), and t-distributed stochastic neighbor embedding (t-SNE) were calculated by Seurat functions: *FindVariableFeatures*, *RunPCA*, *RunUMAP*, and *RunTSNE* (27). The top 2000 variable genes and the top 15 principal components (PCs) were used for UMAP and t-SNE analysis; the number of PCs were selected using “elbow” heuristics. Louvain clustering was performed by Seurat’s *FindClusters* function based on the top 15 PCs, with resolution set to 0.5. Marker gene expression (5–7) and the *Find Markers* function of Seurat was used to calculate gene signatures as overexpressed in the given cell type compared to all other cells. Data were plotted using UMAP, t-SNE, heat maps, dot plots, and violin plots.

##### Identification of differentially expressed genes across cell types

The *FindMarkers* function with the default parameter of the “MAST” method (28) was used to identify differentially expressed genes between treatment groups for each cell type. *FeaturePlot* and *Vlnplot* functions were used to visualize the differentially expressed genes.

##### Gene set enrichment analysis

Gene set enrichment analysis was performed using the fast gene set enrichment analysis (GSEA) package (fgsea v1.18.0) in R (http://bioconductor.org/packages/release/bioc/html/fgsea.html). The R-package implements a novel algorithm to calculate arbitrarily low *P* values quickly and accurately for a collection of gene sets, which allows up to several hundred times faster execution time compared to original Brad implementation of GSEA (29, 30). Hallmark gene sets from Molecular Signature Database (MSigDB; h.all.v7.4.symbols.gmt) were used (31). Gene sets summarize and represent specific well-defined biologic states or processes and display coherent expression. These gene sets were generated by a computational methodology based on identifying overlaps between gene sets in other MSigDB collections and retaining genes that display coordinate expression (31). The Gmt file for hallmark gene sets was downloaded from https://www.gsea-msigdb.org/gsea/msigdb/index.jsp.

#### PL8177 distribution in rats and dogs

The distribution of PL8177 and its main metabolite throughout the GI system was examined in rats and dogs. In the rat study, healthy, male Sprague-Dawley rats (n=12) weighing 250 to 350 g were administered a single 550-µg dose of PL8177 by oral gavage. Rats were sacrificed at 3, 6, or 10 hours postdose (n=4 per time point) to determine PL8177 and the main metabolite levels in the cecum, small intestine, large intestine, colon, and feces using high-performance liquid chromatography.

In a single-dose dog study, 5 healthy, male beagles were given an oral dose of 20 mg PL8177. Dogs were approximately 6 to 7 months old and weighed between 6.4 and 10.6 kg and were housed in groups of up to 3. Blood, urine, and fecal tissue were collected after drug administration. Blood samples were collected on the day of dosing at predose; at 15, 30, and 45 minutes after treatment; and at 1, 2, 4, and 6 hours after treatment. Urine samples for bioanalysis were collected predose for a 24-hour period from each dog and at 0 to 6 hours postdose, and by cystocentesis at necropsy. Fecal samples were collected 2 to 6 hours postdose. Dogs were euthanized 6 hours postdose for evaluation of PL8177 and main metabolite concentrations in the stomach, duodenum, jejunum, ileum, cecum, colon, and rectum.

### Phase 0 clinical study

This was a phase 0, open-label clinical study conducted at a single center in the Netherlands that evaluated the release and absorption of PL8177 and its metabolites in the distal GI tract in healthy male volunteers (n=24) after intake of the oral, solid solution polymer liquid formulation of PL8177. A microdose study design was used to assess whether the oral formulation delivered PL8177 to the appropriate part of the GI tract using a subclinical dose and very small amount of radioactivity.

The objectives of the study were as follows: (1) to demonstrate release of [^14^C]-PL8177 from the polymer solid solution form of [^14^C]-PL8177 in the colon after oral administration through observation of the main metabolite; (2) to confirm that the orally administered, free form of [^14^C]-PL8177 did not result in systemic exposure to [^14^C]-PL8177 and/or its main [^14^C]-metabolite; and (3) to establish the relationship between an oral dose of polymer formulated [^14^C]-PL8177 and the amount of free form [^14^C]-PL8177 and main metabolite in the colon. Inclusion criteria included healthy males of any race; aged 18 to 55 years; body mass index of 18.0 to 30.0 kg/m^2^; no history of irritable bowel syndrome, Crohn’s disease, ulcerative colitis, chronic constipation, or stomach or intestinal surgery or resection that could potentially alter absorption and/or excretion of orally administered drugs; and healthy bowel movements (1–3 per day on average).

A single 70-µg oral dose of PL8177 with a ^14^C radioactive tag was administered to 6 cohorts of 4 adult males each, in the fasted state. Each [^14^C]-PL8177 70-µg oral gelatin capsule contained approximately 35 kBq (0.9 µCi) of radioactivity. Cohorts 1 to 5 received an osmotic laxative (Macrogol [also known as polyethylene glycol] 3350, anhydrous sodium sulfate, sodium chloride, potassium chloride, ascorbic acid, and sodium ascorbate) at 5, 8, 11, 14, or 17 hours postdose, respectively, whereas cohort 6 received no laxative. A laxative was administered at these different time points to ensure excretion of the entire colon contents at different time points. This would, in theory, enable an assessment to be made at different time points, as to whether PL8177 was released from the polymeric oral colon release formulation in the colon. Timing of the laxative administration also allowed for detection of PL8177 and its metabolites in those individuals whose upper GI transit time was >4 hours. Also, if in some individuals the GI transit time was <4 hours from oral administration to colon, it would still be likely that PL8177 and its major metabolite would be identified with the first laxative dose (at 5 hours postdose). After intake of PL8177, cohorts 1 to 5 were monitored for 72 hours postdose, and cohort 6 was monitored for 120 hours postdose.

Safety was monitored and recorded throughout the study *via* adverse event (AE) reporting, laboratory measurements, vital signs, 12-lead electrocardiogram, and physical examination. Blood, urine, and fecal samples were collected at multiple time points daily. Urine was collected with each void and combined into predefined intervals; similarly, feces were collected after each bowel movement and combined into predefined intervals. Standard noncompartmental PK analysis was used to determine concentrations and distribution of PL8177, the main metabolite, and radioactivity in each sample over the study period.

### Statistics

Descriptive statistics were used for assessment of colon damage scores (diarrhea, rectal bleeding, and colon weight gain) (DNBS and DSS rat studies) and for the distribution of PL8177 and main metabolite in the GI tract (Sprague-Dawley rat and dog studies). Mean ± SEM were determined, and 1-way analysis of variance followed by Dunnett’s test was applied for comparison between the vehicle and treated groups. Significance was set at the *P*<0.05 level.

For the phase 0 clinical study, sample sizes were not determined based on statistical considerations but on the number of participants considered sufficient to achieve the study objectives. Descriptive statistics were used for all relevant PK and safety parameters. Statistical analysis and reporting of PK parameters were calculated using Phoenix^®^ WinNonlin^®^ Version 6.3. Additional PK computations were performed using R Version 3.4.0 (R Foundation for Statistical Computing). PK parameters dependent on the terminal rate constant (λz) were not calculated when the adjusted coefficient of determination (*R^2^*) was less than 0.80. PK parameters dependent on area under the concentration-time curve from time 0 to infinity (AUC_∞_) were not calculated when the extrapolated area under the curve (AUC_extra_) was greater than 40%. Renal clearance rate (CL_R_) was calculated for total radioactivity using AUC_0-last_. Because the molecular weights for PL8177 (995.5 g/mol) and its main metabolite (996.5 g/mol) are very similar, the calculations for percentage of dose excreted were conducted without molecular weight corrections.

## Results

### Preclinical studies

#### DNBS colitis–induced rat model of colitis

**Figure 2:**
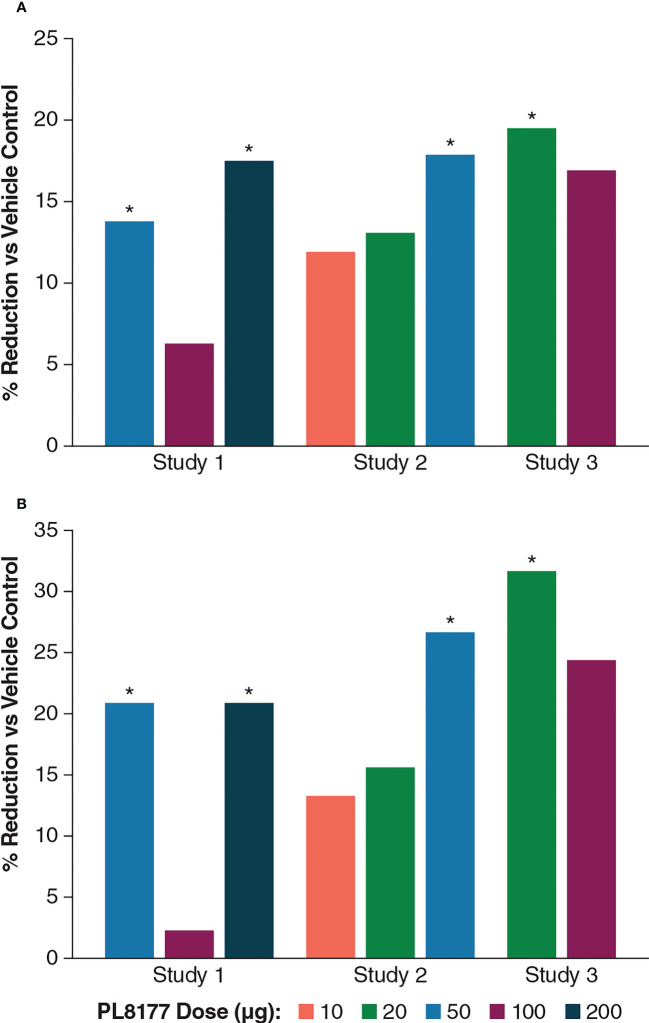
Percentage differences from vehicle across PL8177 doses in DNBS colitis–induced rats: **(A)** macroscopic damage score and **(B)** ulcer/inflammatory score. *P<0.05, treated vs vehicle control; 1-way ANOVA followed by Dunnett’s test. ANOVA, analysis of variance; DNBS, 2,4-dinitrobenzene sulfonic acid.

DNBS induced distal colitis in the rats based on all measured outcomes, including abnormalities of mucosal architecture, extent of inflammation, erosion or ulceration, and epithelial regeneration. As shown in **Table 1**, colonic macroscopic damage scores were reduced in rats treated with PL8177 vs vehicle, with percentage reductions of 11.9% at 10 µg, 13.1% to 19.5% at 20 µg across studies 1 to 3, 13.8% to 17.9% at 50 µg across studies, 6.3% to 16.9% at 100 µg across studies, and 17.5% at 200 µg. More pronounced effects were observed on the ulcer/inflammatory subscore of the macroscopic damage score, with percentage reductions vs vehicle of 13.3% at 10 µg, 15.6% to 31.7% at 20 µg across studies, 20.9% to 26.7% at 50 µg across studies, 2.3% to 24.4% at 100 µg across studies, and 20.9% at 200 µg. Effects of the 50-µg dose of PL8177 on colonic damage (total score and ulcer/inflammatory subscore) were significant (*P*<0.05) in both studies evaluating this dose, with results from studies 1 to 3 shown in **Figure 2**.

**Table 1:** Percentage difference from vehicle in colon observations on day 8 across studies and PL8177 doses in DNBS colitis–induced rats.

Parameter	PL8177 Dose, µg BID							
	Study 1			Study 2			Study 3	
	50	100	200	10	20	50	20	100
Colonic macroscopic damage score	–13.8*	–6.3	–17.5*	–11.9	–13.1	–17.9*	–19.5*	–16.9
Ulcer/inflammatory subscore	–20.9*	–2.3	–20.9*	–13.3	–15.6	–26.7*	–31.7*	–24.4
Stricture score	–10.7	–17.9*	–21.4*	–7.4	–7.4	–3.7	–7.7	–11.5
Net colon-to-body-weight increase^a^	–12.7	–9.3	–19.2*	–23.8	–25.9	–28.1	–33.5*	–24.9
Stool appearance consistency score	–8.7	–4.3	–8.7	–12.0	–12.0	–28.0*	–34.8*	–21.7*
Fecal occult blood score	–11.5	3.8	–7.7	–10.7	–17.9*	–14.3	–8.7	–4.3

All values represent the percentage difference between mean scores in the PL8177 vs vehicle groups.

*P<0.05, treated vs vehicle control; 1-way ANOVA followed by Dunnett’s test.

**^a^** Calculated as the percentage difference between treated and vehicle colon weight/100 g body weight ratio.

ANOVA, analysis of variance; BID, twice daily; DNBS, 2,4-dinitrobenzene sulfonic acid.

Across studies and dose levels, differences from vehicle in colon/body weight ratio increase ranged from -9.3% to -33.5%, and differences from vehicle in stool appearance consistency scores ranged from -4.3% to -34.8% (**Table 1**). Overall, the 20- and 50-µg doses were the most efficacious in this rat model of colitis, which should inform dosing in human studies.

#### DSS colitis–induced rat model of colitis

##### Changes in colitis indices

**Figure 3:**
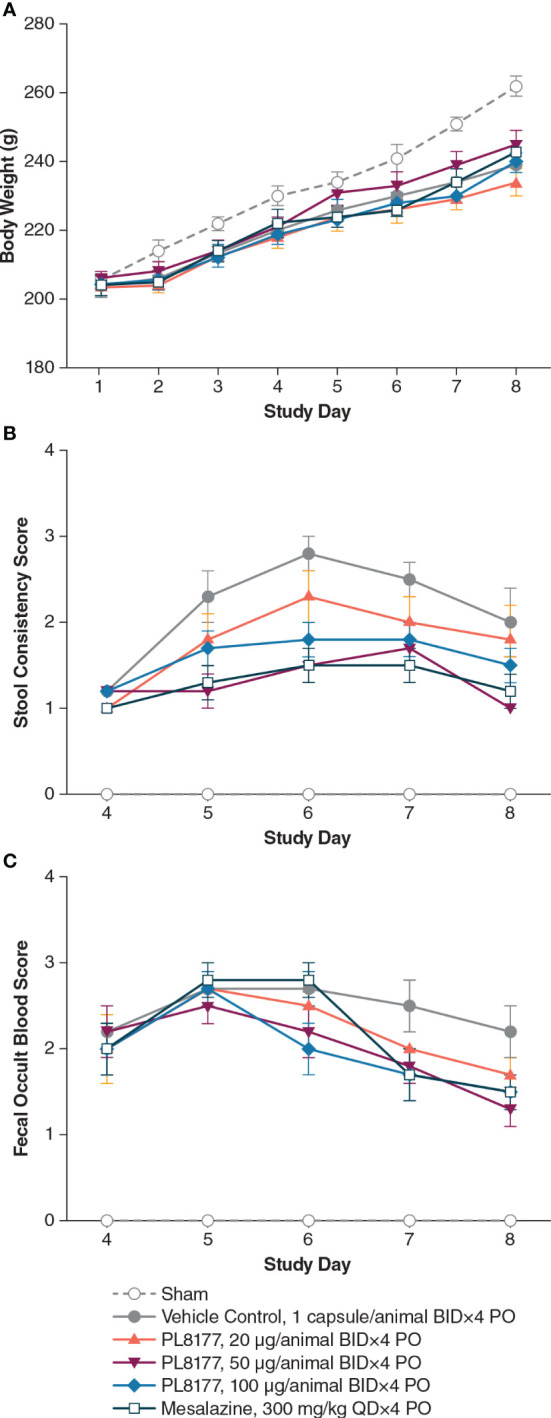
Changes in **(A)** body weight, **(B)** stool consistency, and **(C)** fecal occult blood in DSS colitis–induced rats. Note: all animals, except those in sham group, received 5% DSS in the drinking water for 3 days, from day 1 to day 3, and then changed to normal drinking water for the following 5 days. Tissue harvest occurred on day 8. Data are mean (SEM). BID, twice daily; DSS, dextran sulfate sodium; PO, by mouth; QD, once daily.

DSS in the vehicle group (placebo capsule given orally twice daily for 4 consecutive days, day 4– day 7) caused significant (*P*<0.05) body weight loss from day 7 to day 8 compared to the sham group (no DSS). Body weight gain between the vehicle control and treated groups was similar (**Figure 3A**). The vehicle group also showed significant (*P*<0.05) diarrhea (**Figure 3B**), rectal bleeding (**Figure 3C**), colon weight gain, and colon length shortening (**Table 2**) compared to the sham group, indicating a successful induction of DSS-induced colitis.

**Table 2:** Percentage difference from vehicle for colon weight and length on day 8 across studies and PL8177 doses in DSS colitis–induced rats.

Treatment	Dose/Animal (4-Day Dosing)	Mean Body Weight (SEM) (Day 8), g	Mean Colon Length (SEM), cm	Mean Colon Weight (SEM), g	Mean Colon Weight/100 g Body Weight (SEM)	Net Increase (SEM)	% Decrease in Colon Weight vs Vehicle Control
Sham	N/A	262.3 (3.5)	21.0 (0.4)	0.426 (0.016)	0.162 (0.005)	–	–
Vehicle control (placebo)	1 capsule	239.2* (3.3)	16.8* (0.6)	0.655* (0.018)	0.274* (0.008)	0.112* (0.008)	–
PL8177	20 μg BID	234.0 (3.6)	15.3 (0.4)	0.568 (0.022)	0.243 (0.012)	0.081 (0.012)	28
PL8177	50 μg BID	245.3 (4.0)	17.4 (0.3)	0.528^†^ (0.029)	0.215^†^ (0.012)	0.053^†^ (0.012)	53
PL8177	100 μg BID	240.3 (2.9)	16.0 (0.5)	0.565 (0.023)	0.235 (0.009)	0.073 (0.009)	35
Mesalazine	300 mg/kg QD	242.5 (3.0)	18.6 (0.5)	0.570 (0.031)	0.235 (0.012)	0.073 (0.012)	35

BID, twice daily; DSS, dextran sulfate sodium; N/A, not applicable; QD, once daily.

*P<0.05 vs sham. ^†^ *P*<0.05 vs vehicle control.

PL8177, when given orally at 20, 50, and 100 μg/animal twice daily for 4 days, resulted in inhibition of colon weight gain (mean reductions of 28%, 53%, and 35%, respectively) when compared with the vehicle group (placebo capsule). The PL8177 50-μg twice-daily dose showed a significant (*P*<0.05) 53% reduction (improvement) in colon weight in the study compared with the vehicle (**Table 2**). Mesalazine at 300 mg/kg orally once daily for 4 consecutive days was associated with moderate improvement in colon weight gain (35% decrease) and marked reduction in colon length shortening when compared with the vehicle group.

PL8177 at 50 μg/animal showed significant (*P*<0.05) improvement in stool consistency score from day 5 to day 8 and significant (*P*<0.05) improvement in fecal occult blood score on day 8 when compared with the vehicle group (**Figures 3B, C** **)**. Moreover, PL8177 when given orally at 100 μg/animal, had a significant (*P*<0.05) effect on stool consistency score on day 6. With PL8177 50 μg/animal, there was a moderate decrease in colonic IL-6 cytokine level after harvesting on day 8 vs the vehicle group (mean [SEM] 159.8 [30.8] pg/mL vs 241.3 [40.7] pg/mL), but little or no effect on colonic IL-1β, TNF-α, or IFN--γ cytokine levels.

##### Histologic assessment

**Figure 4:**
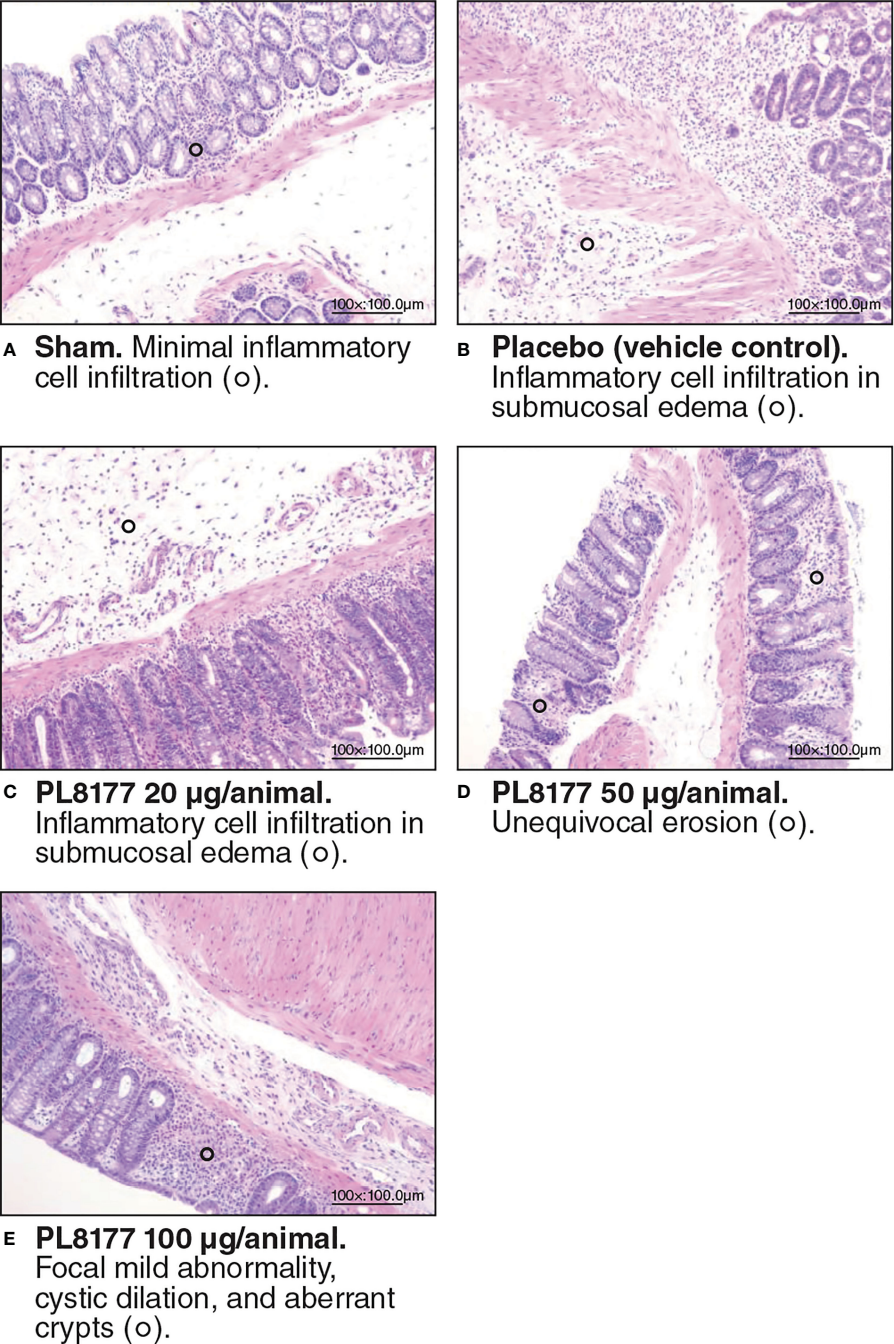
Representative colon histologic sections of DSS colitis–induced rats. Sham **(A)** is no challenge and no treatment. Placebo/vehicle control **(B)** is no treatment but DSS challenge. **(C-E)** are DSS challenge and treatment with PL8177. DSS, dextran sulfate sodium.

**Figure 5:**
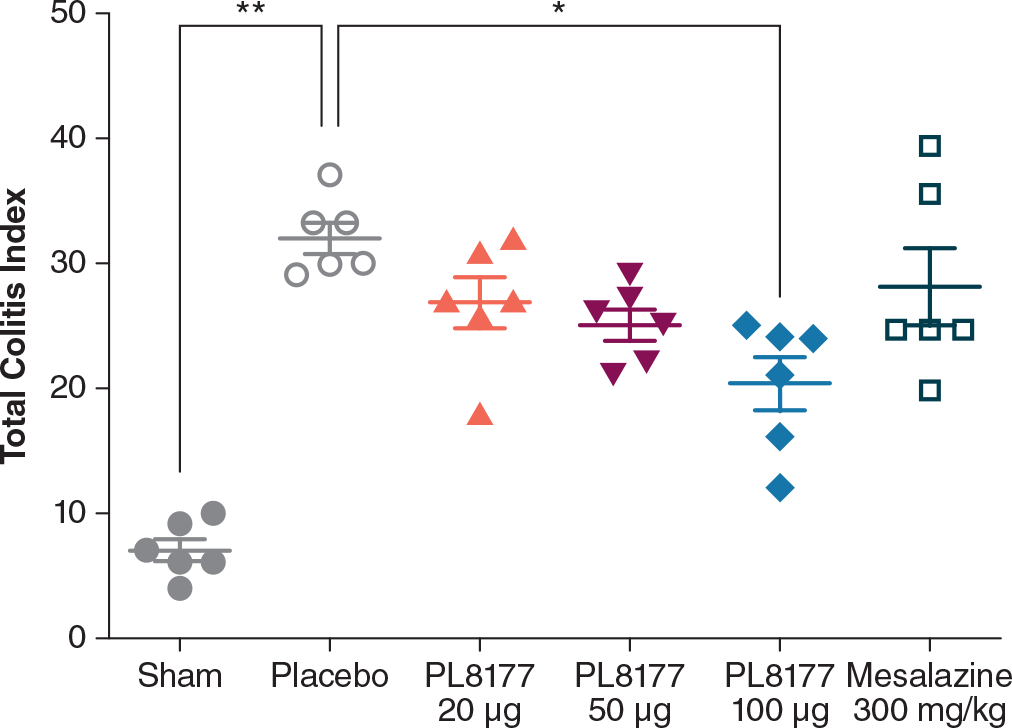
Total colitis index for DSS colitis–induced rat model. Data shown are mean ( ± SEM) and individual data points. *P<0.05. **P<0.01 one-way ANOVA followed by Dunnett’s test. ANOVA, analysis of variance, DSS, dextran sulfate sodium.

Histologic assessment of distal colon from vehicle-treated rats revealed mild to severe transmural disruption of the normal architecture of the colon. Focal ulceration, disrupted crypt architecture, pronounced focal to multifocal inflammatory cell infiltrate, and mild to moderate transmural intestinal thickening were apparent in histologic sections of colon (**Figure 4**). Colon histopathologic examination showed injury and prominent ulcerations to the mucosa of the distal colon extended for 2.5 to 7 cm in treated rats. Typically, focal edema in the submucosa and pronounced transmural thickening of the colonic wall were also noted. The total colitis lesion score, an assessment that included separate items of severe diffuse mucosal architectural abnormalities, ulceration, crypt dilation, aberrant crypts, crypt loss, distortion of mucosal glands, goblet cell loss, and focal regeneration of the epithelium, was assessed in all treatment groups. There was a significant (*P*<0.05) improvement observed in the total colitis index with PL8177 100 μg/animal vs vehicle control (mean [SD] total colitis score 20.3 [5.2] n=6 vs 32.0 [3.0] n=6, respectively; **Figure 5**). The decline in the mean total colitis index for mesalazine-treated rats was less than that observed in any of PL8177-treated rats (mean [SD] score of 28.5 [7.71]; n=6). PL8177 was well tolerated at the tested dose levels, and no overt toxicities were observed during the study period.

##### Immunochemistry

**Figure 6:**
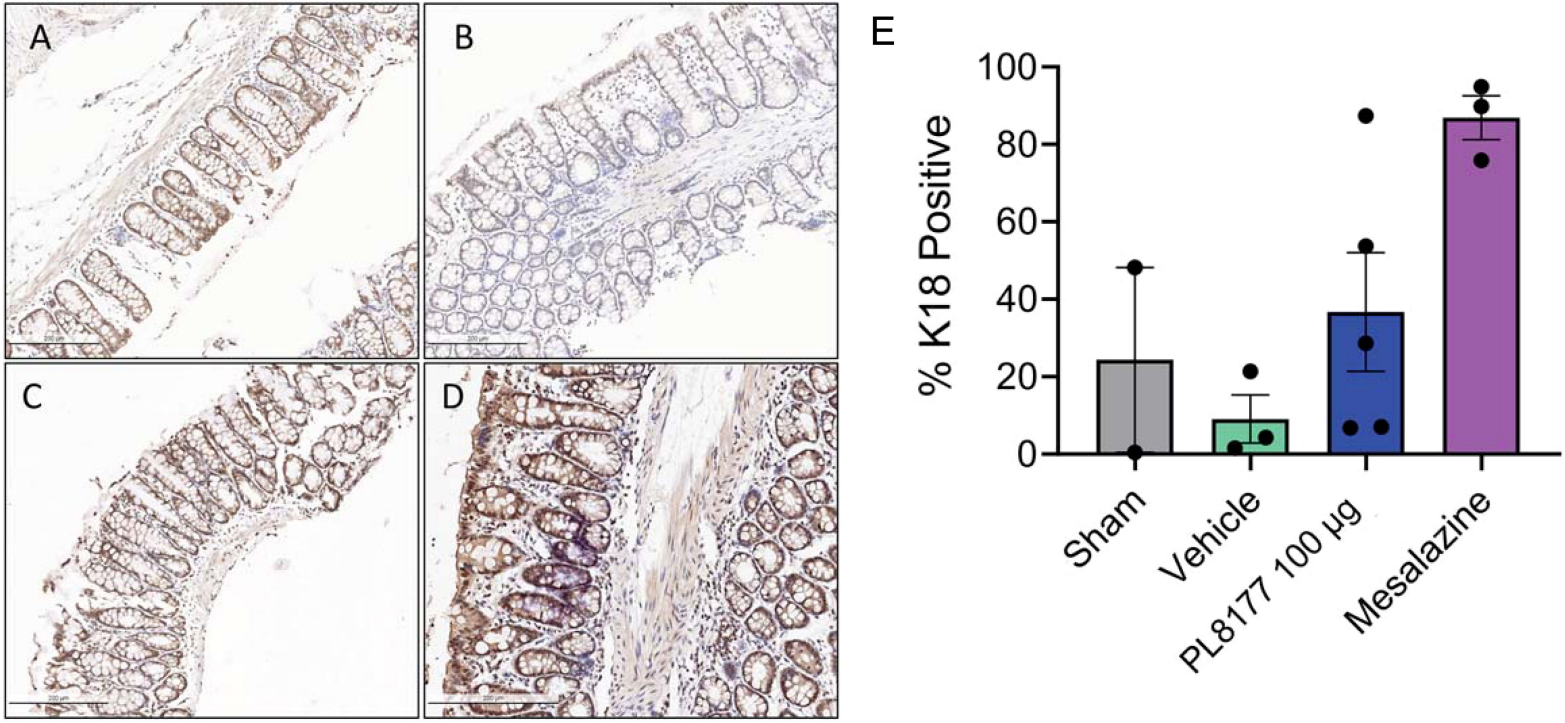
Representative colon immunohistochemistry for K18 sections of DSS colitis-induced rats. Sham **(A)** is no challenge and no treatment. Placebo/vehicle control **(B)** is no treatment but DSS challenge. **(C)** is DSS challenge and treatment with PL8177 (100 µg). **(D)** is DSS challenge and treatment with mesalazine. **(E)** Quantification of positive K18 cells in colon crypts versus total crypt cells. Notes: material was limited for sham staining resulting in 2 colon tissues. Bar chart shows mean, SEM, and data points. DSS, dextran sulfate sodium; K18, cytokeratin 18.

Cytokeratin 18 (K18) is a known marker of epithelial cells and enterocytes in the colon (32). Immunohistochemistry for K18 was performed in the rat colons from the DSS rat model. There was a decreased number of K18 positive cells in the vehicle colons compared to sham (**Figure 6**). Treatment with PL8177 or mesalazine resulted in an increased number of K18 positive cells, similar to sham. As a note, material was limited for immunohistochemistry resulting in staining for only 2 sham colons.

##### Single nuclei profiling of diseased, healthy, and treated rat colon tissues

**Figure 7:**
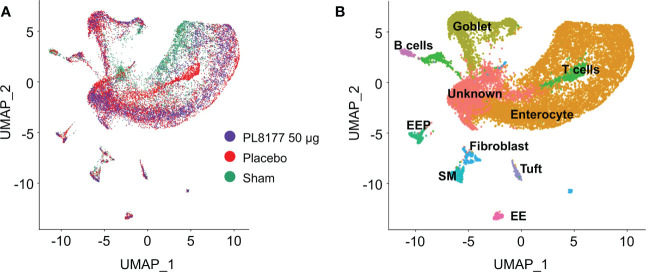
Single nuclei RNA-seq analysis reveals cellular composition of DSS colitis–induced rat colon tissue in sham, placebo, and PL8177 50 µg samples. **(A)** UMAP plots display cell-type clusters derived from all the treatment groups. Each dot represents a cell. Cells in PL8177 50 µg samples are shown in violet, placebo in red, and sham in green. **(B)** UMAP visualization of cells after annotating cell clusters. Expression of cell-type marker genes was used to annotate each cluster. Colors of the dots represent the cluster to which the cells belong. DSS, dextran sulfate sodium; EE; enteroendocrine; EEP, enteroendocrine progenitor; SM, smooth muscle; UMAP, Uniform Manifold Approximation and Projection.

**Figure 8:**
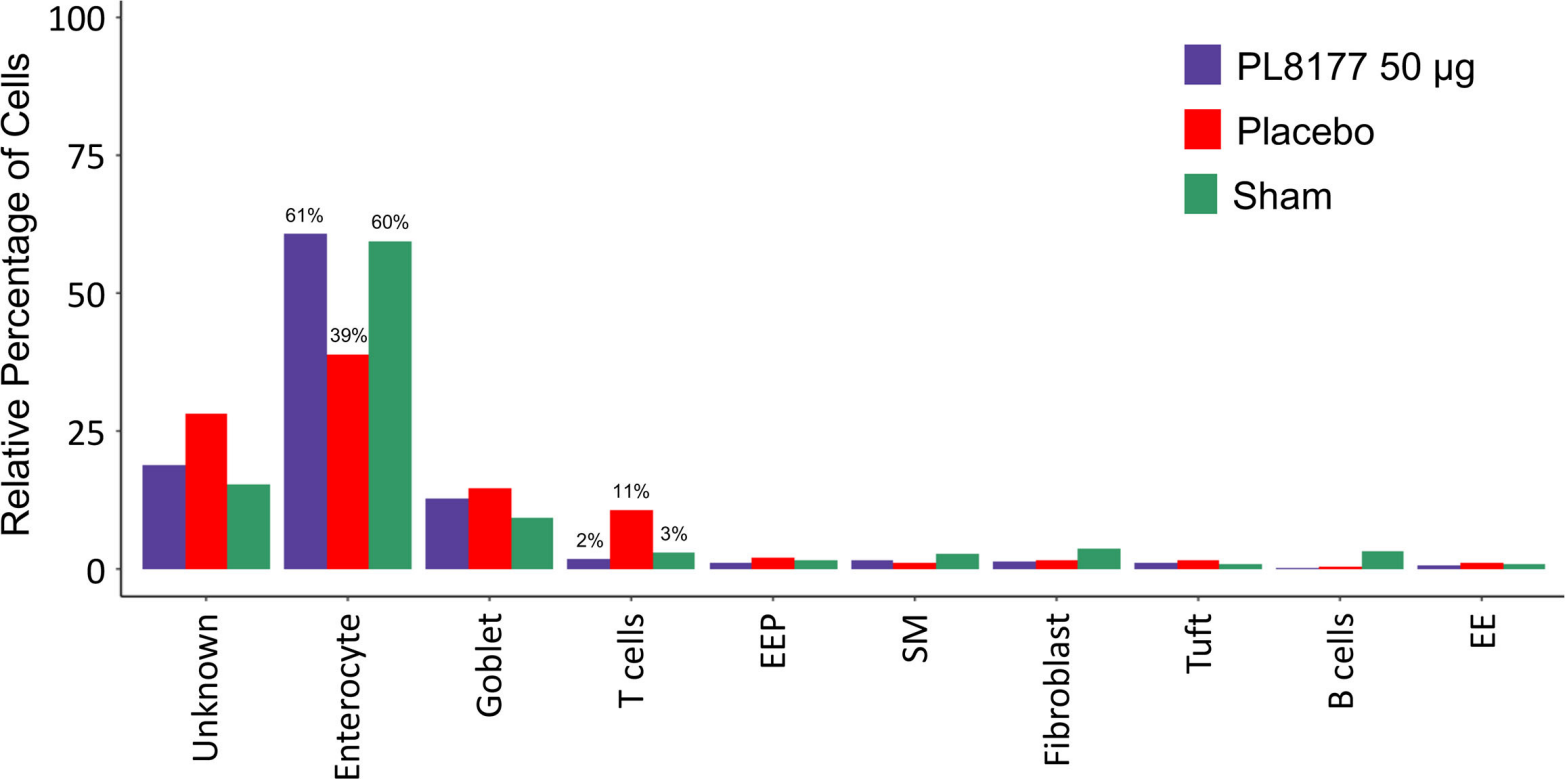
Relative cell type percentage in different treatment groups of DSS colitis–induced rats. PL8177 50 µg treated samples show a relative increase in the number of enterocytes and decrease in T cells compared to placebo. DSS, dextran sulfate sodium; EE, enteroendocrine; EEP, enteroendocrine progenitor; SM, smooth muscle.

To identify cellular level changes in the transcriptomic profiles of healthy, diseased, and treated rats, colon tissue was obtained from sham, placebo, and PL8177 (50 µg dose) treated animals (n=3 per treatment type). Colon samples were collected and subjected to snRNA-seq, using the 10x Genomics chromium platform. On average, 1885 nuclei and 13,954 genes per sample, with a total of 17,388 genes and 17,258 nuclei across all treatments were obtained after quality controlled filtering and used for downstream analysis. Data from each treatment were merged and unsupervised UMAP analysis was performed to visualize clustering of single cells from all treatment groups (**Figure 7A**). We used expression of canonical genes (see Methods) to annotate clusters into 9 cell types: enterocyte, goblet, tuft, B cells, T cells, enteroendocrine, enteroendocrine progenitor, smooth muscle, and fibroblast (**Figure 7B**, **Figure S1**). Clusters missing marker gene expression were annotated as “Unknown.” Based on the number of nuclei in each cluster, we calculated the relative percentage of nuclei for each cell type in the sham, placebo, and PL8177 50 µg treatment groups. As shown in **Figure 8**, the composition of cell types from different treatment groups was generally consistent between treatments, except for enterocytes, T cells, and B cells, which have the most pronounced differences. Enterocytes are the predominant epithelial cell type in the lumen of small intestine and colon (33, 34). Several studies have reported excessive shedding of ileal and colonic epithelium and loss of barrier integrity under the high inflammation milieu found in ulcerative colitis (35–39). We observed loss of enterocyte cells in the placebo group, with a drop in relative cell type percentage from 60% in sham to 39% in placebo. Another indication of increased inflammatory insult in the placebo group is a high relative percentage of T cells. The placebo group had 11% of the total cells as T cells, while in the sham group we observed 3% of the total cells were T cells. Treatment of rats with PL8177 50 µg showed significant increase in the proportion of enterocytes (61%) and a decrease in the proportion of T cell relative percentage (2%). We also observed a high percentage of Cd19+ B cells in sham compared to placebo and PL8177 50 µg treated samples.

##### Transcriptionally distinct T cell and enterocyte population in PL8177 vs placebo groups

**Figure 9:**
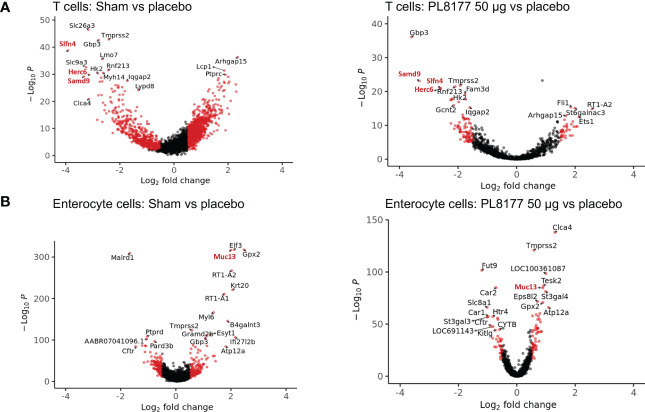
Genes differentially expressed in T cells and enterocyte cells of sham and PL8177 50 µg DSS colitis–induced rats as compared to placebo group. **(A)** Differentially expressed genes in T cells. Shown in red are genes with log_2_FC >1.5 and *P*<0.00001. Gene names in red are the top common differentially expressed genes in sham and PL8177 50 µg T cells. **(B)** Differentially expressed genes in enterocytes. Shown in red are genes with log_2_FC >0.5 and *P*<0.001. The gene name in red is the top common differentially expressed gene in sham and PL8177 50 µg enterocyte cells.

**Figure 10:**
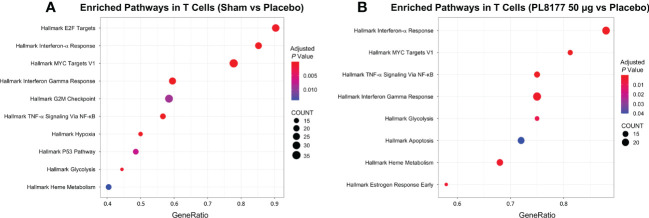
Enrichment analysis for differentially expressed T cell cluster genes. Dot plot shows the top 10 significant pathways enriched in T cells of sham **(A)** and PL8177 50 µg **(B)** DSS colitis–induced rats. Differentially expressed genes in sham and PL8177 50 µg as compared to placebo were used for enrichment analysis. Dot size indicates the gene ratio (enriched genes/total number of genes). Dot color indicates the adjusted *P* value for enrichment analysis. Count indicates number of enriched genes in each pathway. DSS, dextran sulfate sodium; NF-κB, nuclear factor kappa B; TNF-α, tumor necrosis factor alpha.

Differential gene expression analysis was performed to compare the transcriptome of T cells and enterocytes of the different treatment groups (**Figure 9**). In total, 204 and 115 genes were significantly differentially expressed in the T cells of sham vs placebo animals and PL8177 50 µg vs placebo animals, respectively (adjusted *P* value <0.05 and abs log_2_FoldChange [log_2_FC] >1.5). Sixty-three common genes were significantly downregulated in the T cells of sham and PL8177 animals as compared to placebo (**Figure S1**, **Table S2**). Two hundred and fifty-five and 95 genes were significantly differentially expressed in enterocytes (adjusted *P* value <0.05 and abs log_2_FC >0.5), with 32 common upregulated genes, in sham vs placebo and PL8177 50 µg vs placebo, respectively. Unlike T cells, we observe more genes to be significantly upregulated in enterocytes of sham and PL8177 animals (**Figure 9B**, **Table S3**). *Samd9* and *Herc6* were among the top 10 common significantly downregulated T cell genes with a −3.3- and −3.1-fold change (sham vs placebo, respectively) and −3.2- and −2.6-fold change (PL8177 50 µg vs placebo, respectively). *Muc13* was one of the top genes with significantly high expression in enterocytes of sham (log_2_FC:2.0) and PL8177 (log_2_FC:0.9) animals. To identify pathways associated with these differentially expressed genes, we performed GSEA (29, 40) with the MSigDB hallmark gene set (31) using clusterProfiler (41). GSEA analysis showed negative enrichment of multiple immune pathways in the T cells of the sham and PL8177 50 µg groups (**Figure 10**). Common top negatively enriched pathways in the T cells of sham and PL8177 50 µg groups include IFN-α, IFN-γ signaling, and TNF signaling *via* NF-kB.

#### PL8177 distribution in rats and dogs

**Figure 11:**
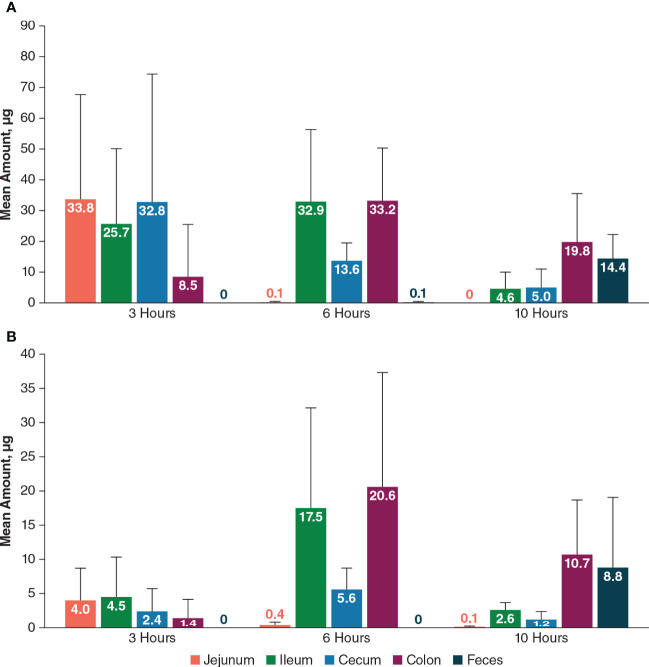
Mean amount of PL8177 **(A)** and its main active metabolite **(B)** in the GI tract of rats at 3, 6, and 10 hours postdose after a single oral 550-µg dose. GI, gastrointestinal.

PL8177 levels after an orally administered dose of 550 µg in rats were highest in jejunum, ileum, and cecum at 3 hours (25.7 µg –33.8 µg) and were highest in the colon at 6 hours (33.2 µg) and 10 hours (19.8 µg; **Figure 11**). Low levels of the main metabolite (≤4.0 µg) were detected in the small intestine (jejunum and ileum) at 3 hours and 10 hours; the highest levels were detected in the ileum and colon at 6 hours and in the colon and feces at 10 hours.

**Figure 12:**
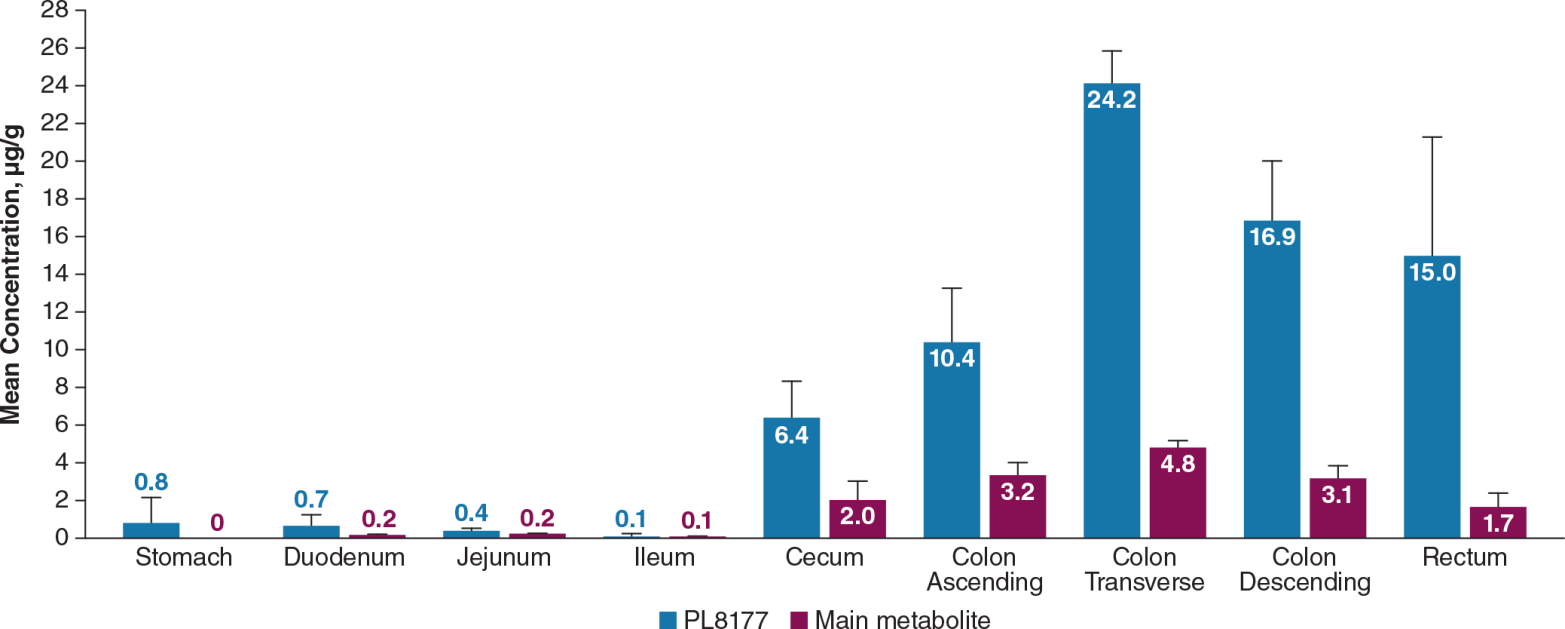
Mean concentration of PL8177 and the main metabolite in the GI tract of dogs at 6 hours after a single oral 20-mg dose. Levels of main metabolite in stomach were below the limit of quantification in all samples. GI, gastrointestinal.

In dogs, mean total PL8177 concentrations at 6 hours after a single oral dose were very low (≤0.8 µg/g) in upper GI tract tissues (stomach, duodenum, jejunum, ileum); the highest concentrations were observed in the transverse colon, descending colon, rectum, ascending colon, and cecum (mean ± SEM, 24.2 ± 1.7, 16.9 ± 3.2, 15.0 ± 6.3, 10.4 ± 2.9, and 6.4 ± 1.9 µg/g, respectively; **Figure 12**). Concentrations of the main metabolite were lower in all tissues compared with PL8177 but were similarly proportional, with very low levels detected in the upper GI tract. The percentage of the total oral PL8177 dose in the colon at 6 hours (4.2%) was ≥22-fold greater than the percentage observed across upper GI tract organs. PL8177 and the main metabolite concentrations in plasma and urine were below the lower limit of quantification and were not detected in all samples; fecal concentrations could not be measured.

### Phase 0 clinical study

A total of 24 healthy male participants were enrolled in the study. The median age was 30 years (range, 18–55 years), and the majority (83%) were white.

#### Pharmacokinetics

As expected, no [^14^C]-PL8177 or [^14^C]-metabolite was found in plasma and urine samples; therefore, no PK parameters could be calculated for these analytes. In plasma and urine, the majority of radioactivity was identified as [^14^C]-phenylalanine, an amino acid component of [^14^C]-PL8177. Radioactivity was measurable but low in plasma beginning 1 hour after administration and was not affected by laxative. The geometric mean maximum concentration (C_max_) was 0.6 to 0.8 ng eq/mL across cohorts with geometric percentage coefficient of variation (CV%) for C_max_ ranging from 11.3% to 59.1% (**Table 3**). Individual time to maximum concentration (t_max_) values varied widely, with a range from 2 to 36 hours across all cohorts. AUC_0-t_ values were consistent among cohorts 1 through 5 (in which participants received a laxative) and larger for cohort 6 (no laxative). Because sampling was extended from 72 to 120 hours for cohort 6, this difference was anticipated. The geometric mean of total radioactivity half-life (t_½_) in plasma was roughly 100 hours for cohorts 1 through 5, but was approximately double for cohort 6. This difference could also be an artifact of the longer sampling in this cohort. Because of the long estimated t_½_ values across all cohorts compared to the duration of sampling, caution is warranted when evaluating these values.

**Table 3:** Plasma PK parameters for total radioactivity in human participants by cohort in phase 0 clinical study.

Cohort	Dose		C_max,_ mean (CV%), ng eq/mL	t_max,_ mean (range), h	AUC_0-t_, mean (CV%), h*ng eq/mL	t_½_, mean (CV%), h	CL_R_, mean (CV%), L/h
1	[^14^C]-PL8177 + laxative 5 h postdose	n	4	4	4	3	4
			0.626 (16.4)	21.02 (2.00, 36.00)	34.9 (23.6)	111 (16.7)	0.306 (61.8)
2	[^14^C]-PL8177 + laxative 8 h postdose	n	4	4	4	4	4
			0.830 (24.7)	4.96 (2.00, 12.00)	48.1 (26.4)	119 (2.01)	0.319 (15.9)
3	[^14^C]-PL8177 + laxative 11 h postdose	n	4	4	4	3	4
			0.742 (11.3)	12.01 (11.92, 24.00)	42.0 (8.44)	96.9 (6.29)	0.408 (27.5)
4	[^14^C]-PL8177 + laxative 14 h postdose	n	4	4	4	4	4
			0.762 (20.4)	12.00 (12.00, 18.00)	45.2 (19.1)	111 (3.34)	0.356 (13.0)
5	[^14^C]-PL8177 + laxative 17 h postdose	n	4	4	4	4	4
			0.644 (59.1)	17.93 (17.92, 17.95)	36.4 (61.0)	118 (11.7)	0.486 (41.3)
6	[^14^C]-PL8177 without laxative	n	4	4	4	4	4
			0.687 (19.2)	10.00 (2.00, 18.08)	57.9 (15.2)	207 (8.53)	0.347 (19.2)

AUC_0-t_, area under the plasma concentration-time curve from time 0 to time t; CL_R_, renal clearance of drug from plasma; C_max_, maximum plasma concentration; CV%, geometric percentage coefficient of variation; PK, pharmacokinetic; t_½_, elimination half-life; t_max_, time to reach C_max_.

**Figure 13:**
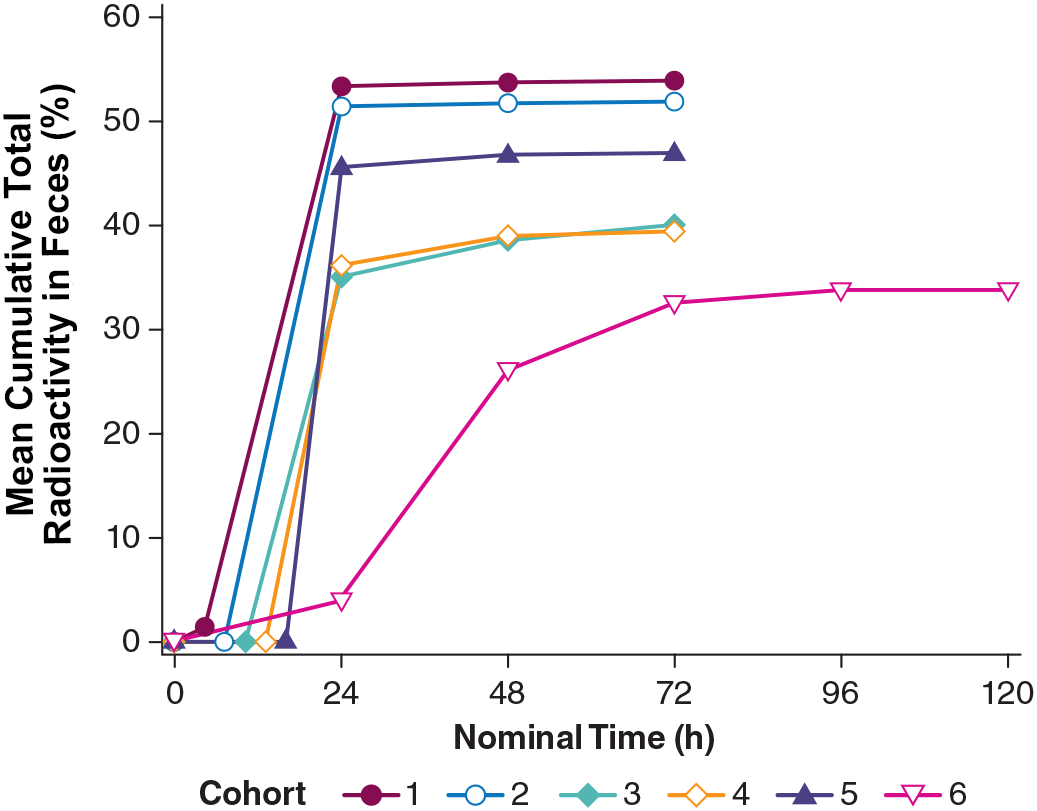
Total radioactivity in feces excreted by healthy humans across cohorts from the phase 0 clinical study. Cohort 1 = [^14^C]-PL8177 + laxative 5 h postdose; Cohort 2 = [^14^C]-PL8177 + laxative 8 h postdose; Cohort 3 = [^14^C]-PL8177 + laxative 11 h postdose; Cohort 4 = [^14^C]-PL8177 + laxative 14 h postdose; Cohort 5 = [^14^C]-PL8177 + laxative 17 h postdose; and Cohort 6 = [^14^C]-PL8177 without laxative.

Radioactivity in urine exhibited a mean cumulative percentage of dose excreted (PE) of 16.5% to 29.0% across cohorts up to 72 hours postdose. [^14^C]-PL8177 was detected in the feces of 12 out of 24 participants for at least 1 time interval, and the maximum mean cumulative PE was <3.0% for all cohorts. [^14^C]-metabolite was detected in the feces of 10 participants and had a maximum mean cumulative PE of <2% for all cohorts. Fecal radioactivity was recorded at all time points and had a mean cumulative PE of 33.9% to 53.9% across cohorts (**Figure 13** **)**. On average, in participants who received a laxative, 40%–50% of total radioactivity was recovered in feces. In participants who did not receive a laxative, approximately 30% of radioactivity was recovered in feces. From this, we conclude that the maximum time frame of 72 or 120 hours is insufficient to allow for excretion of all of the dosed radioactivity, or that the remaining radioactivity was cleared *via* the urine or exhalation. In cohort 6 (no laxative) the cumulative percentage of total radioactivity in the feces reached a plateau at slightly higher than 30% after 120 hours. This suggests that PL8177 or peptide fragments were retained in the GI tract where they were extensively metabolized over an extended period of time of >5 days.

#### Safety

A summary of treatment-emergent AEs (TEAEs) by treatment group and severity is shown in **Table 4**. In participants who received both [^14^C]-PL8177 and laxative, reported TEAEs occurring in >10% of participants were abdominal pain, diarrhea, flatulence, nausea, vomiting, fatigue, and dizziness, which are consistent with the known AEs of the laxative used. There was one moderate severity TEAE of nausea, which started 30 minutes after treatment with the laxative, lasted approximately 6.5 hours, and did not require any concomitant medication. No TEAEs were considered related to [^14^C]-PL8177. Two participants who received PL8177 without laxative reported TEAEs of abdominal pain, headache, and erythema (1 participant had 1 TEAE and 1 participant had 2); all were mild in severity and judged not be related to PL8177. None of the reported TEAEs led to discontinuation of participants from the study. There were no severe TEAEs. Overall, there were no trends or clinically significant changes observed in clinical laboratory parameters, vital signs, 12-lead electrocardiograms, or physical examinations.

**Table 4:** Summary of TEAEs and severity in humans.

Treatment (No. of Participants)	AEs					
	Mild		Moderate		Overall	
	Events, n	Participants, n (%)	Events, n	Participants, n (%)	Events, n	Participants, n (%)
Total number of AEs (n=24)	37	16 (67)	1	1 (4)	38	16 (67)
[^14^C]-PL8177 (n=4)	3	2 (50)			3	2 (50)
[^14^C]-PL8177 + laxative at 5 h postdose (n=4)	7	3 (75)			7	3 (75)
[^14^C]-PL8177 + laxative at 8 h postdose (n=4)	7	3 (75)			7	3 (75)
[^14^C]-PL8177 + laxative at 11 h postdose (n=4)	4	3 (75)	1	1 (25)	5	3 (75)
[^14^C]-PL8177 + laxative at 14 h postdose (n=4)	12	4 (100)			12	4 (100)
[^14^C]-PL8177 + laxative at 17 h postdose (n=4)	4	1 (25)			4	1 (25)

AE, adverse event; TEAE, treatment-emergent adverse event.

There were no TEAEs related to [^14^C]-PL8177, and therefore these are all unrelated to treatment. There were no severe AEs.

## Discussion

This paper examined an oral formulation of the selective MC1R agonist PL8177 in rat models of colitis and evaluated the distribution and PK of orally delivered PL8177 in preclinical animal models and in healthy male volunteers. Previously published work assessed PL8177 in a proof-of-principle study in a rectally cannulated DNBS rat colitis model versus vehicle and oral sulfasalazine. Intracolonically injected PL8177 was significantly superior to untreated controls at doses of 0.5, 1.5, and 5.0 µg per rat when corrected for vehicle (*P*<0.05) in reducing bowel inflammation parameters, effects similar to sulfasalazine (17). Another paper investigated the PK of PL8177 after subcutaneous delivery in mice, rats, dogs, and healthy humans (18). The measured exposure levels resulted in pharmacologically active PL8177 concentrations at the targeted MC1R. Rapid absorption was seen in healthy volunteers with detectable plasma levels observed within 15 min after a single dose and multiple-dose administration over 7 days resulted in pharmacokinetic characteristics similar to those after single-dose administration. Subcutaneously or intracolonically administered PL8177 cannot be directly compared with the oral administration presented in this paper. The data in previous papers are similar in a qualitative manner to the current data in this paper, within normal expected experimental variation.

In the DNBS rat model of colitis, significant protective effects of 50 µg oral polymer-encapsulated PL8177 were observed across multiple outcomes of colon damage. In the DSS rat model of colitis, administration of PL8177 50 µg resulted in significant improvements in colon weight gain, stool consistency, and fecal blood score compared with the vehicle control group at day 8. Notably, these outcomes were not dose dependent, with no consistent improvement in outcomes at higher doses. Histologic examination of DSS rats showed that PL8177 also reduced the total colitis index score (a measure of colitis inflammation and therefore the pharmacodynamic efficacy of PL8177) when compared to the placebo vehicle. Compared with administration of mesalazine, PL8177 showed a greater reduction in colon weight and greater decline in mean total colitis index. Improvements in stool consistency and fecal occult blood by PL8177 treatment in DSS rats also suggest efficacy in limiting colitis in this model.

The results of the preclinical studies and the phase 0 clinical study show the bioavailability of PL8177 within the lower GI tract in rats, dogs, and humans. PL8177 was detected in much higher proportions in the colon compared with the upper GI tract in rats and dogs. In humans, neither the parent drug [^14^C]-PL8177 nor its metabolite [^14^C]-metabolite was quantifiable in plasma and urine, demonstrating the lack of systemic circulation of the polymer formulation of PL8177 or its metabolite. The appearance of radioactivity in plasma approximately 1 hour after administration is consistent with the release of PL8177 from the polymer matrix in the small intestine and degradation into absorbable amino acids. Urinary recovery of total radioactivity was approximately 20% to 30% of the total dose; fecal elimination of total radioactivity was approximately 30% in participants who did not receive a laxative and 40% to 50% in those who received laxative.

When delivered orally, unprotected PL8177 is thoroughly metabolized before reaching the colon. The conversion of PL8177 to the main metabolite occurs when PL8177 is in free form (not polymer bound) and is therefore available (Palatin Technologies, Inc. data on file). Thus, the presence of the main metabolite is indicative of the release of PL8177 from the polymer and its bioavailability within the colon. The results of the human microdose study demonstrate that PL8177 or the metabolite (PL8435) is not absorbed systemically when released in the GI tract, which suggests that systemic side effects should not be observed. There were no safety or tolerability issues associated with the microdose of PL8177 in the clinical study, and the safety results showed that the majority of AEs were associated with laxative use. The dose of PL8177 used in the clinical trial, however, was very small (70 µg), and extrapolating these safety results to therapeutic doses is not warranted. Taken together, these results demonstrate that PL8177 was released from the polymer formulation and extensively metabolized within the GI tract. The resulting inactive amino acid fragments of the original peptide were absorbed across the intestinal lumen. This explanation is supported by the fact that the only observable radioactivity in plasma corresponded to [^14^C]-labeled phenylalanine.

MC1Rs are present on the epithelial cells of the colon lumen (15), and previous studies examining topical application of PL8177 *via* cannula in a disease model of IBD have demonstrated potent efficacy (17). Specifically, PL8177 significantly reduced bowel inflammation at various doses compared with placebo controls, with greater effect sizes at higher doses. The results of the present study are in accordance with Spana et al. and other findings from animal models that have demonstrated a benefit of α-MSH in mitigating inflammatory disease processes, including colitis (16, 17, 42–45). In a murine model of acute and chronic colitis, twice-daily α-MSH exposure was associated with an 80% reduction in fecal blood, reduced weight loss, and reduced TNF-α in the lower colon and nitric oxide production in the lower bowel (16). Similarly, Váradi et al. showed that administration of α-MSH resulted in protection against barrier disruption in human Caco-2 epithelial cell lines with barrier damage induced by TNF-α and IL-1β (45).

PL8177 and its main metabolite are selective MC1R agonists mimicking the activity of α-MSH. Moreover, MC1R has been shown to have a functional role in GI inflammation (15). Given that α-MSH is a highly potent anti-inflammatory neuropeptide, promising findings like those from the current study support ongoing investigation into whether MC1R agonist therapies can yield the same benefits as α-MSH and possibly prevent or reverse inflammation in GI and other organ systems. Although the role of α-MSH in controlling localized and systemic inflammation has been established (11), comparatively less research has focused on selective MC1R agonists as therapeutic options for IBD. The current study underscores the importance of further investigations to clarify the possible value of melanocortin peptide candidate drugs for IBD. The research presented here using the DSS rat model suggests that the DSS treatment caused a loss in colon enterocyte cells, which has been observed as a characteristic of IBD in other studies (39). DSS treatment also increased the relative proportions of T and Cd19+ B cells in the colon, markers of inflammation. After PL8177 treatment the relative number of enterocytes were increased and the proportion of T and Cd19+ B cells were reduced in comparison to animals on placebo, in agreement with the improvement of the markers of colitis observed with the other histologic measures (colon weight reduction, stool consistency, and fecal occult blood score). The proportion of enterocytes in the PL8177 50 µg treated group was similar to that of the sham (non DSS-treated) group, 61% and 62%, respectively. The proportion of T cells was 3% in the sham and 2% in the PL8177 50 µg group, compared to 11% in the placebo group.

Differential gene expression analysis showed that *Samd9* and *Herc6* were significantly downregulated T cell genes in sham and PL8177 50 µg-treated colons. Both these genes are members of the IFN-α response pathway, a well-studied inflammatory pathway known to be dysregulated in autoimmune disease (46, 47). In enterocytes, *Muc13* was one of the top genes with significantly higher expression in the sham and PL8177 50 µg groups. *Muc13* is a transmembrane mucin, highly expressed in the enterocyte cells of the small and large intestine (48). It is known to protect against inflammation by inhibiting epithelial cell apoptosis (49). Sheng et al. have shown that loss of *Muc13* in DSS challenged mice caused severe and acute colitis (49). IFN-α, IFN-γ signaling, and TNF signaling *via* NF-κB were negatively enriched pathways in the T cells of sham and PL8177 50 µg groups. IFN-α, IFN-γ, and TNF are characteristic of IBD (39, 50, 51), so negative enrichment of these pathways suggests a role in reducing inflammation. Increased K18 positive enterocytes in PL8177-treated DSS colon tissue validates results from snRNA-seq and suggests maintenance of key cell structures to protect the colon barrier. Together, these findings support the histopathology results and suggest reduced immune infiltration, resolution of inflammation, and maintenance of an intact colon structure and barrier with oral PL8177 50 µg treatment.

The findings of the clinical study should be interpreted in light of some limitations. Preclinical and phase 0/early phase 1 clinical trials are important first steps in assembling foundational evidence about a potential therapeutic but are not designed to address efficacy in humans. Furthermore, the variability of dose responses observed across studies elucidate the need for additional studies consisting of larger sample sizes to reduce the variability inherent in these animal models. Also, the small sample size and the very low drug exposure mean that serious AEs and rare events are less likely to be detected. Future studies of longer duration with higher, therapeutic doses will be necessary to fully evaluate the safety profile of PL8177. In a phase 1 study that evaluated the safety and tolerability of a subcutaneously injected formulation of PL8177 in healthy volunteers, however, there were no reports of serious or severe AEs owing to PL8177 and none that led to discontinuation. The most commonly reported AEs in that study were injection related (injection-site pain, erythema, and induration) and skin hyperpigmentation (18). Documented side effects from other melanocortin receptor ligand clinical candidates include transient increases in blood pressure and nonserious skin reactions (eg, hyperpigmentation) (52). Finally, findings from preclinical studies may not be generalizable to humans with inflammatory GI conditions—a target population for this candidate drug—and additional research in larger populations is needed to better identify the full range of anti-inflammatory and other clinical benefits of PL8177 in humans with IBD.

In conclusion, these results show that the selective MC1R agonist candidate drug PL8177 was successfully delivered to the lower GI tract with the expected safety and tolerability characteristics when administered orally as a polymer-bound formulation. The observed reduction in colonic damage and inflammation compared with vehicle treatment in rats is consistent with its aim of ultimately treating IBD in humans. The single nuclei profiling, gene expression analyses, and immunochemistry are consistent with a mechanism of action to be anticipated by a selective MC1R agonist. These findings support the continued development and investigation into PL8177 for the treatment of IBD.

## Data availability statement

The data presented in the study are deposited in the National Center for Biotechnology Information, Gene Expression Omnibus repository, accession number GSM6964554. Available below https://www.ncbi.nlm.nih.gov/geo/query/acc.cgi?acc=GSM6964554.

## Ethics statement

The studies involving human participants were reviewed and approved by Ethics Committee of Beoordeling Ethiek Biomedisch Onderzoek (Assen, Netherlands). The patients/participants provided their written informed consent to participate in this study. The animal study was reviewed and approved by Institutional Animal Care and Use Committee at Pharmacology Discovery Services Taiwan, Ltd. ITR Laboratories Canada Inc. (Baie d’Urfe, QC, Canada), Animal Care Committee.

## Author contributions

All authors listed have made a substantial, direct, and intellectual contribution to the work and approved it for publication.

## Supplementary material

Supplementary Figure 1 Marker gene expression for each cell type of DSS colitis–induced rats, where dot size represents percentage of cells within a cluster with marker gene expression (percent expressed) and color encodes the average expression level across all the cells within a cluster (average expression). The plot is an integrated PL8177 50 µg, sham, and placebo Seurat object. Three samples from each group. DSS, dextran sulfate sodium; EE, enteroendocrine; EEP, enteroendocrine progenitor; SM, smooth muscle.
