# Supplementary material for: A novel oral formulation of the melanocortin-1 receptor agonist PL8177 resolves inflammation in preclinical studies of inflammatory bowel disease and is gut restricted in rats, dogs, and humans
Source: Front Immunol. 2023 Feb 20;14:1083333. doi: 10.3389/fimmu.2023.1083333 (PMC9986545; doi:10.3389/fimmu.2023.1083333)
Supplement: Supplementary file 1 [file Table_1.pdf]

Table S1. Scoring Table for Calculating Total Colitis Index Score in DSS Colitis–Induced Rats

Item	Description	Score
Abnormalities of mucosal architecture	None (Normal)	0
	Minimal for focal, not exceeding laminar propria	1
	Mild abnormality, cystic dilation/aberrant crypts	2
	Moderate or multifocal abnormalities	3
	Severe, entire crypt and epithelium lost	4
Extent of inflammation	None	0
	Minimal for focal, scattered calls (<10%)	1
	Mild (10%–25%)	2
	Moderate, inflammatory cells extending into the submucosa	3
	Severe, transmural leukocytic infiltrate from mucosa to serosa	4
Erosion or ulceration	No erosion, ulceration, or granulation tissue	0
	Minimal or focal, not exceeding lamina propria	1
	Unequivocal erosion	2
	Moderate ulceration	3
	Severe ulceration or granulation tissue	4
Epithelial regression	Complete regeneration or normal tissue	0
	Almost complete regeneration	1
	Regeneration with crypt depletion	2
	Surface epithelium not intact	3
	No tissue repair	4
Involvement, %	1–25	1
	26–50	2
	51–75	3
	76–100	4

Note: Total colitis index was derived by summing 3 sections per animal. Total colitis index range: 0–60.
