# Supplementary material for: A novel oral formulation of the melanocortin-1 receptor agonist PL8177 resolves inflammation in preclinical studies of inflammatory bowel disease and is gut restricted in rats, dogs, and humans
Source: Front Immunol. 2023 Feb 20;14:1083333. doi: 10.3389/fimmu.2023.1083333 (PMC9986545; doi:10.3389/fimmu.2023.1083333)
Supplement: Supplementary file 2 [file Table_2.pdf]

Table S2. Common Significantly Downregulated Genes in T Cells of Sham and PL8177 50 µg DSS Colitis–Induced Rats as Compared to Placebo

Gene	*P* value (Sham vs Placebo)	Avg_log$_2$FC (Sham vs Placebo)	pct.1 (Sham vs Placebo)	pct.2 (Sham vs Placebo)	*P* value adj (Sham vs Placebo)	*P* value (PL8177 vs Placebo)	Avg_log$_2$FC (PL8177 vs Placebo)	pct.1 (PL8177 vs Placebo)	pct.2 (PL8177 vs Placebo)	*P* value adj (PL8177 vs Placebo)
AABR07025140.1	3.55024E-57	-3.972158743	0.242	0.759	6.17316E-53	1.00731E-32	-2.868205185	0.146	0.759	1.75151E-28
AABR07025272.1	2.10087E-21	-2.998559757	0.05	0.415	3.65299E-17	1.82395E-15	-2.707543103	0.042	0.415	3.17148E-11
AABR07059663.1	5.7428E-12	-1.953116012	0.125	0.365	9.98558E-08	9.14601E-15	-2.441128671	0.021	0.365	1.59031E-10
AC135826.1	5.55982E-26	-1.913932367	0.317	0.655	9.66742E-22	2.35356E-18	-2.211638488	0.219	0.655	4.09237E-14
Actn4	3.75092E-23	-2.011797368	0.35	0.632	6.52211E-19	1.30231E-12	-1.736987753	0.292	0.632	2.26446E-08
Agmo	3.11731E-20	-2.622561237	0.167	0.443	5.42038E-16	2.36235E-09	-1.912999866	0.167	0.443	4.10766E-05
Atp10b	1.25451E-19	-1.874753442	0.325	0.608	2.18134E-15	1.63321E-12	-1.629745518	0.26	0.608	2.83983E-08
Cdhr2	7.42194E-10	-1.575506782	0.1	0.347	1.29053E-05	8.19128E-09	-1.732666018	0.073	0.347	0.00014243
Clic5	2.69644E-16	-2.500670303	0.092	0.395	4.68857E-12	9.59426E-09	-1.934467462	0.146	0.395	0.000166825
Cobl	1.14708E-20	-2.339544127	0.175	0.496	1.99455E-16	3.36437E-10	-1.70564876	0.229	0.496	5.84996E-06
Cwh43	3.40451E-13	-1.921543908	0.175	0.451	5.91977E-09	2.05318E-10	-1.994897302	0.188	0.451	3.57007E-06
Dhrs9	1.95443E-13	-2.073643974	0.008	0.253	3.39836E-09	2.08809E-06	-1.521506245	0.052	0.253	0.03630766
Egfr	1.01103E-11	-1.994099642	0.142	0.314	1.75798E-07	3.08021E-07	-1.818898792	0.083	0.314	0.005355866
Eps8	2.76972E-24	-2.256872504	0.258	0.584	4.81599E-20	3.21343E-11	-1.568440576	0.25	0.584	5.58751E-07
Eps8l2	1.66236E-29	-2.693831744	0.15	0.582	2.89051E-25	1.92921E-12	-1.795491884	0.26	0.582	3.35451E-08
Erbb3	1.82096E-07	-1.534064292	0.058	0.203	0.003166292	4.8008E-08	-1.678491392	0.01	0.203	0.000834764
Fam13b	2.77961E-21	-1.906517269	0.333	0.621	4.83319E-17	1.0415E-12	-1.672064391	0.281	0.621	1.81095E-08
Fam160a1	1.40323E-10	-1.55657891	0.083	0.347	2.43994E-06	2.42016E-08	-1.664637777	0.083	0.347	0.000420817
Fam3d	1.75422E-24	-1.814989485	0.408	0.8	3.05024E-20	6.7511E-20	-1.761353001	0.396	0.8	1.17388E-15
Gbp3	2.29538E-43	-2.780876864	0.242	0.774	3.99121E-39	8.43128E-37	-3.591229007	0.177	0.774	1.46603E-32
Gcnt2	3.10832E-23	-1.881220469	0.383	0.641	5.40474E-19	1.29847E-16	-2.147036816	0.271	0.641	2.25777E-12
Herc6	1.70712E-30	-3.133355911	0.125	0.552	2.96834E-26	1.58165E-21	-2.616133868	0.083	0.552	2.75016E-17
Hk2	4.39112E-31	-2.81802057	0.333	0.709	7.63529E-27	1.34571E-18	-2.206836084	0.312	0.709	2.33992E-14
Iqgap2	2.68518E-28	-1.721691345	0.567	0.749	4.66898E-24	5.14558E-16	-1.602127032	0.396	0.749	8.94714E-12
Krt20	4.57367E-21	-2.03226951	0.2	0.596	7.95269E-17	1.5934E-11	-1.620380101	0.24	0.596	2.77061E-07
Lmo7	2.28439E-36	-2.651989689	0.267	0.662	3.9721E-32	1.23123E-17	-1.973912646	0.26	0.662	2.14086E-13
Lrrc1	1.34821E-13	-1.818605776	0.192	0.372	2.34427E-09	1.54724E-06	-1.501293943	0.177	0.372	0.026903427
Magi1	5.20589E-14	-1.585308983	0.342	0.485	9.05199E-10	9.49719E-08	-1.540948126	0.25	0.485	0.001651371
Malrd1	2.40784E-19	-2.543824232	0.058	0.445	4.18675E-15	2.2518E-08	-1.62900354	0.177	0.445	0.000391543
March3	2.04857E-17	-2.56685954	0.208	0.47	3.56206E-13	2.76947E-07	-1.626495626	0.26	0.47	0.004815557
Mep1a	2.77496E-12	-2.047495675	0.058	0.322	4.82509E-08	1.14635E-07	-1.509702793	0.073	0.322	0.001993277
Mier3	1.16763E-10	-1.903241371	0.108	0.304	2.03027E-06	2.62274E-07	-1.729259842	0.073	0.304	0.004560417
Mtmr11	1.06794E-12	-1.909422135	0.167	0.384	1.85694E-08	1.37538E-07	-1.693639739	0.135	0.384	0.002391508
Myh14	3.02766E-31	-2.619341462	0.225	0.582	5.2645E-27	4.64347E-13	-1.825616144	0.198	0.582	8.07407E-09

adj, adjusted; Avg, average; DSS, dextran sodium sulfate; pct.1, percentage of cells where feature is detected in first group; pct.2, percentage of cells where the feature is detected in the second group; Sham vs Placebo, genes differentially expressed in sham as compared to placebo; PL8177 vs Placebo, genes differentially expressed in PL8177 50 µg vs placebo.

Gene	*P* value (Sham vs Placebo)	Avg_log$_2$FC (Sham vs Placebo)	pct.1 (Sham vs Placebo)	pct.2 (Sham vs Placebo)	*P* value adj (Sham vs Placebo)	*P* value (PL8177 vs Placebo)	Avg_log$_2$FC (PL8177 vs Placebo)	pct.1 (PL8177 vs Placebo)	pct.2 (PL8177 vs Placebo)	*P* value adj (PL8177 vs Placebo)
Oasl2	1.73528E-15	-1.719427054	0.242	0.531	3.01731E-11	3.08532E-11	-1.739957527	0.198	0.531	5.36475E-07
Parp9	6.59926E-17	-1.970030353	0.217	0.45	1.14748E-12	1.32567E-11	-1.952611218	0.115	0.45	2.30508E-07
Pde9a	1.11023E-14	-2.052260996	0.15	0.45	1.93047E-10	8.75689E-09	-1.668694939	0.167	0.45	0.000152265
Phlpp2	2.8684E-17	-2.158913711	0.15	0.478	4.98758E-13	6.45059E-10	-1.777520531	0.167	0.478	1.12163E-05
Pik3c3	3.54647E-12	-1.750998506	0.183	0.379	6.16661E-08	8.32133E-09	-1.935389943	0.104	0.379	0.000144691
Plekha7	2.28849E-17	-1.897574195	0.217	0.488	3.97923E-13	1.37668E-10	-1.773838928	0.198	0.488	2.39377E-06
Ppp2r3a	4.38343E-11	-1.822035307	0.117	0.324	7.62191E-07	6.36489E-08	-1.626618106	0.073	0.324	0.001106728
Ptprh	4.2741E-17	-2.269793783	0.092	0.398	7.43181E-13	9.12849E-09	-1.824673906	0.156	0.398	0.000158726
RGD1624210	4.00177E-08	-1.532324727	0.025	0.212	0.000695827	3.28101E-07	-1.626237047	0.021	0.212	0.005705029
Rnd3	1.37549E-08	-1.768416059	0.108	0.256	0.00023917	2.01683E-06	-1.59505713	0.052	0.256	0.035068608
Rnf213	3.01759E-32	-2.431833335	0.308	0.724	5.24699E-28	3.63705E-22	-2.098434374	0.281	0.724	6.3241E-18
Rock2	1.00999E-26	-2.160649271	0.358	0.63	1.75617E-22	7.17907E-14	-1.839855165	0.312	0.63	1.2483E-09
Rsad2	1.32919E-10	-2.064733527	0.008	0.213	2.3112E-06	2.72108E-07	-1.782047804	0.021	0.213	0.004731409
Samd9	7.31522E-30	-3.328175888	0.033	0.525	1.27197E-25	7.90946E-24	-3.321361606	0.031	0.525	1.3753E-19
Sectm1b	3.94773E-09	-1.921396099	0.033	0.238	6.86431E-05	1.05766E-09	-2.130246705	0.01	0.238	1.83906E-05
Slc26a2	4.73144E-17	-2.40133494	0.15	0.41	8.22703E-13	2.74255E-11	-2.24280369	0.104	0.41	4.76874E-07
Slc26a3	3.30709E-47	-3.160675992	0.317	0.791	5.75037E-43	2.01413E-18	-1.72891384	0.375	0.791	3.50216E-14
Slc9a2	1.57261E-16	-2.254820265	0.142	0.433	2.73446E-12	4.19388E-10	-1.930793364	0.135	0.433	7.29232E-06
Slc9a3	2.92482E-33	-3.248891815	0.108	0.618	5.08567E-29	3.52066E-18	-2.253178862	0.198	0.618	6.12173E-14
Slfn4	1.63593E-39	-3.914662685	0.317	0.738	2.84455E-35	1.05217E-21	-2.587208516	0.51	0.738	1.82951E-17
Stk25	9.16194E-17	-2.096926677	0.175	0.426	1.59308E-12	2.50611E-08	-1.501477187	0.146	0.426	0.000435762
Tmc5	2.94244E-18	-2.175103042	0.2	0.471	5.11632E-14	1.33519E-08	-1.582274487	0.188	0.471	0.000232163
Tmem236	7.76592E-13	-2.034911036	0.108	0.378	1.35034E-08	9.47046E-09	-1.729800777	0.104	0.378	0.000164672
Tmem45b	8.6231E-19	-2.039743494	0.283	0.615	1.49938E-14	3.56643E-10	-1.548957431	0.292	0.615	6.2013E-06
Tmprss2	1.23847E-43	-2.410055531	0.475	0.838	2.15345E-39	1.55139E-22	-1.930587333	0.49	0.838	2.69756E-18
Trim31	1.82678E-12	-2.107627691	0.075	0.343	3.1764E-08	1.40269E-09	-1.947064546	0.062	0.343	2.43899E-05
Trpm6	1.52488E-17	-2.224597199	0.167	0.42	2.65147E-13	3.85716E-10	-2.018277503	0.135	0.42	6.70682E-06
Txn1	5.56519E-23	-2.790141344	0.1	0.475	9.67675E-19	3.35747E-09	-1.582780471	0.229	0.475	5.83797E-05
Vil1	1.51193E-13	-1.792498107	0.175	0.401	2.62895E-09	1.19139E-10	-2.094793467	0.115	0.401	2.0716E-06

adj, adjusted; Avg, average; DSS, dextran sodium sulfate; pct.1, percentage of cells where feature is detected in first group; pct.2, percentage of cells where the feature is detected in the second group; Sham vs Placebo, genes differentially expressed in sham as compared to placebo; PL8177 vs Placebo, genes differentially expressed in PL8177 50 µg vs placebo.
