# Supplementary material for: A novel oral formulation of the melanocortin-1 receptor agonist PL8177 resolves inflammation in preclinical studies of inflammatory bowel disease and is gut restricted in rats, dogs, and humans
Source: Front Immunol. 2023 Feb 20;14:1083333. doi: 10.3389/fimmu.2023.1083333 (PMC9986545; doi:10.3389/fimmu.2023.1083333)
Supplement: Supplementary file 3 [file Table_3.pdf]

Table S3. Common Significantly Upregulated Genes in Enterocyte Cells of Sham and PL8177 50 μg DSS Colitis–Induced Rats as Compared to Placebo

Gene	*P* value (Sham vs Placebo)	Avg_log$_2$FC (Sham vs Placebo)	pct.1 (Sham vs Placebo)	pct.2 (Sham vs Placebo)	*P* value adj (Sham vs Placebo)	*P* value (PL8177 vs Placebo)	Avg_log$_2$FC (PL8177 vs Placebo)	pct.1 (PL8177 vs Placebo)	pct.2 (PL8177 vs Placebo)	*P* value adj (PL8177 vs Placebo)
AABR07000398.1	1.67845E-86	1.12883652	0.982	0.96	2.9185E-82	4.20535E-26	0.665813221	0.975	0.96	7.31227E-22
AC135826.1	2.01282E-63	1.063822616	0.639	0.531	3.49988E-59	6.11859E-32	0.522681567	0.63	0.531	1.0639E-27
Apaf1	3.70622E-25	0.792342601	0.289	0.215	6.44438E-21	1.09163E-46	0.726661038	0.362	0.215	1.89812E-42
Aqp8	1.65341E-28	0.655814504	0.409	0.339	2.87495E-24	1.16599E-43	0.838444314	0.466	0.339	2.02742E-39
Atp12a	4.91954E-85	1.808283848	0.395	0.203	8.55409E-81	1.18932E-66	1.084814307	0.397	0.203	2.068E-62
Ceacam1	6.49306E-42	1.002991206	0.216	0.132	1.12901E-37	2.32209E-43	0.833089633	0.249	0.132	4.03765E-39
Clca4	2.81817E-37	0.727029881	0.469	0.359	4.90024E-33	9.9962E-139	1.303977347	0.625	0.359	1.7381E-134
Clic5	1.78706E-18	0.647582611	0.31	0.274	3.10734E-14	1.4137E-41	0.670963015	0.414	0.274	2.45814E-37
Esyt1	1.0953E-113	1.236492838	0.522	0.376	1.9045E-109	1.58775E-47	0.648422435	0.508	0.376	2.76078E-43
Gpx2	0	2.469885082	0.825	0.461	0	1.63919E-71	0.895722067	0.648	0.461	2.85023E-67
Itpk1	2.37198E-24	0.554275754	0.343	0.314	4.12439E-20	5.30963E-47	0.708976846	0.458	0.314	9.23239E-43
Krt20	1.7015E-221	2.058321499	0.778	0.546	2.9587E-217	3.48774E-58	0.738790254	0.675	0.546	6.06449E-54
Lnx2	1.38482E-26	0.582450054	0.356	0.343	2.40793E-22	7.97699E-29	0.549685202	0.458	0.343	1.38704E-24
LOC100361087	6.26813E-34	0.65517498	0.389	0.327	1.0899E-29	3.3771E-99	0.992786033	0.518	0.327	5.87218E-95
Mapk14	6.56804E-55	0.941023016	0.445	0.364	1.14205E-50	4.31644E-26	0.51393915	0.461	0.364	7.50543E-22
Muc13	0	2.00570305	0.862	0.667	0	1.72543E-85	0.891681378	0.784	0.667	3.00017E-81
Nectin2	4.13185E-15	0.61367611	0.251	0.205	7.18446E-11	4.91877E-22	0.51492851	0.301	0.205	8.55275E-18
Osbpl3	3.58493E-17	0.572751206	0.26	0.227	6.23348E-13	6.60144E-34	0.644810272	0.341	0.227	1.14786E-29
Plac8	8.56516E-40	0.763606324	0.418	0.328	1.48931E-35	2.68332E-55	0.778558154	0.484	0.328	4.66576E-51
Plcd3	2.3593E-16	0.717659636	0.214	0.166	4.10236E-12	4.09419E-43	0.751942631	0.299	0.166	7.11897E-39
Plekha2	3.09413E-24	0.886447487	0.252	0.18	5.38008E-20	4.13778E-18	0.504421255	0.265	0.18	7.19478E-14
Plekhg6	1.66328E-24	0.80263442	0.22	0.165	2.89211E-20	1.12844E-25	0.545703254	0.264	0.165	1.96213E-21
Ripk4	1.03825E-17	0.654270862	0.218	0.148	1.80531E-13	2.22634E-30	0.591444688	0.26	0.148	3.87117E-26
RT1-CE10	2.98475E-24	0.639946815	0.305	0.267	5.18989E-20	9.0933E-18	0.597898705	0.337	0.267	1.58114E-13
Scnn1a	7.43447E-40	0.87544485	0.333	0.27	1.29271E-35	9.51568E-37	0.636027928	0.389	0.27	1.65459E-32
Shroom3	4.25926E-69	0.529485206	0.668	0.666	7.406E-65	1.43345E-58	0.534668918	0.746	0.666	2.49249E-54
Sort1	1.69174E-17	0.561474248	0.312	0.295	2.94159E-13	7.62801E-28	0.527541749	0.415	0.295	1.32636E-23
St3gal4	1.74605E-51	0.834786192	0.345	0.304	3.03603E-47	5.26097E-82	0.986172631	0.481	0.304	9.14777E-78
Stard13	4.8768E-24	0.616217141	0.339	0.268	8.47978E-20	7.02969E-43	0.713225181	0.407	0.268	1.22232E-38
Tesk2	5.17825E-75	0.888328158	0.494	0.403	9.00395E-71	1.38069E-87	0.915453618	0.57	0.403	2.40075E-83
Tmprss2	6.5426E-124	0.576777613	0.841	0.838	1.1376E-119	1.2872E-121	0.582053985	0.903	0.838	2.2382E-117
Trpv6	2.57809E-16	0.616184898	0.24	0.219	4.48278E-12	3.04679E-34	0.764159182	0.324	0.219	5.29776E-30

adj, adjusted; Avg, average; DSS, dextran sodium sulfate; pct.1, percentage of cells where feature is detected in first group; pct.2, percentage of cells where the feature is detected in the second group; Sham vs Placebo, genes differentially expressed in sham as compared to placebo; PL8177 vs Placebo, genes differentially expressed in PL8177 50 μg vs placebo.
