# Supplementary figures and images for: A novel oral formulation of the melanocortin-1 receptor agonist PL8177 resolves inflammation in preclinical studies of inflammatory bowel disease and is gut restricted in rats, dogs, and humans
Source: Front Immunol. 2023 Feb 20;14:1083333. doi: 10.3389/fimmu.2023.1083333 (PMC9986545; doi:10.3389/fimmu.2023.1083333)

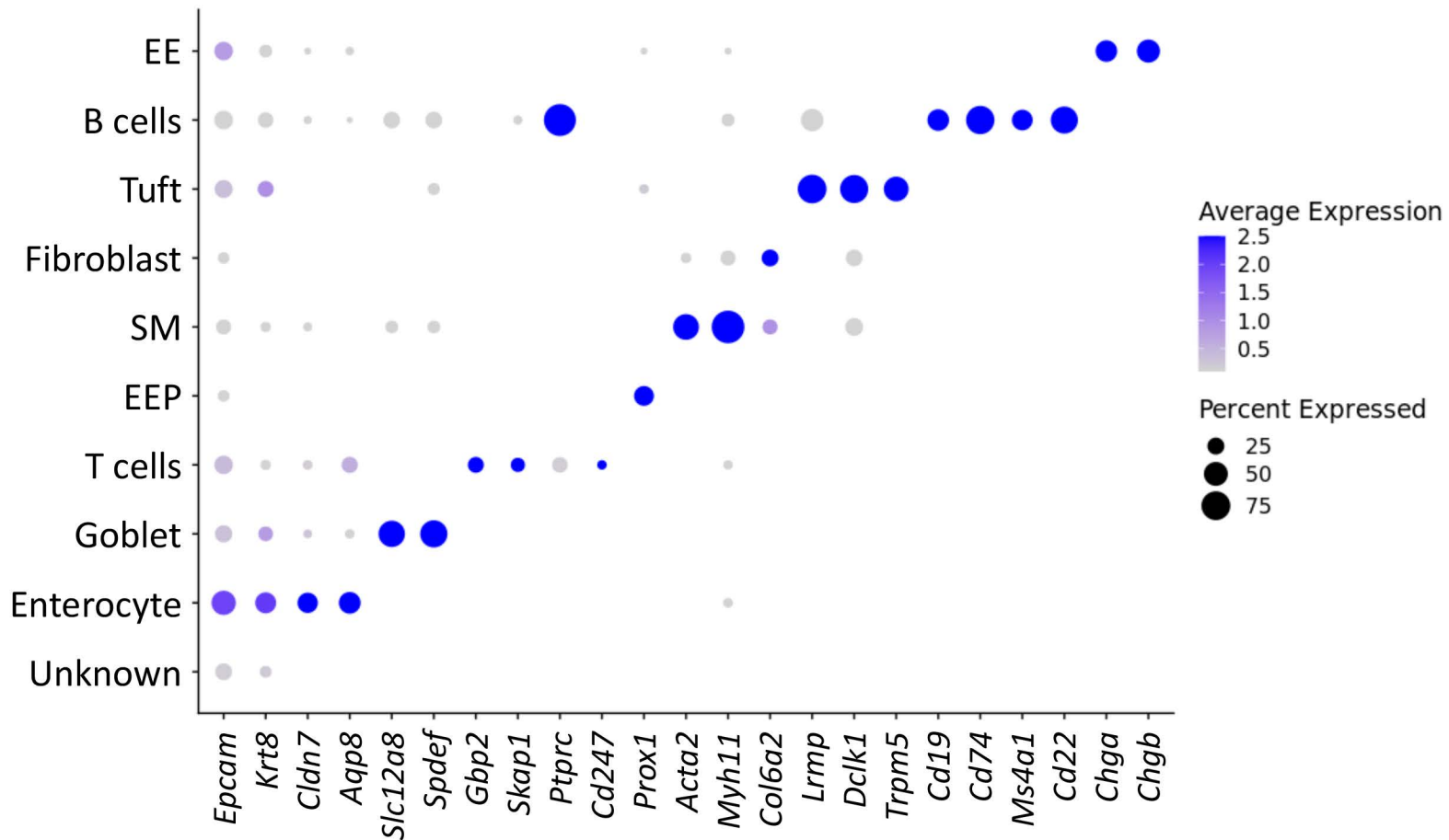

Supplement: Supplementary Figure 1 — Marker gene expression for each cell type of DSS colitis–induced rats, where dot size represents percentage of cells within a cluster with marker gene expression (percent expressed) and color encodes the average expression level across all the cells within a cluster (average expression). The plot is an integrated PL8177 50 µg, sham, and placebo Seurat object. Three samples from each group. DSS, dextran sulfate sodium; EE, enteroendocrine; EEP, enteroendocrine progenitor; SM, smooth muscle. [file Image_1.pdf]
